# In Silico Analysis Uncovers FOXA1 as a Potential Biomarker for Predicting Neoadjuvant Chemotherapy Response in Fine-Needle Aspiration Biopsies

**DOI:** 10.7150/jca.101901

**Published:** 2024-09-30

**Authors:** Zhenglang Yin, Jianfei Tao, Yanyan Liu, Haohao Chen, Kongwang Hu, Yao Wang, Maoming Xiong

**Affiliations:** 1 Department of General surgery, The First Affiliated Hospital of Anhui Medical University, Hefei, 230022, China.; 2 Department of General surgery, The Third Affiliated Hospital of Anhui Medical University, Hefei, 230061, China.; 3 Department of General surgery, The People's Hospital of Feidong County, Hefei, 231699, China.; 4 Department of Thoracic Surgery, The People's Hospital of Feidong County, Hefei, 231699, China.; 5 Digestive Endoscopy Department, Jiangsu Province Hospital, The First Affiliated Hospital with Nanjing Medical University, Nanjing, 210029, China.

**Keywords:** Breast cancer, Machine learning, Biomarker, Genomics, Bioinformatics

## Abstract

**Background:** The preoperative identification of neoadjuvant chemotherapy (NAC) treatment responsiveness in breast cancer (BC) patients is advantageous for tailoring treatment regimens. There is a relative scarcity in the current research exploring NAC treatment responsive biomarkers using bulk sequencing data obtained from fine-needle aspiration (FNA).

**Materials and Methods:** Limma was employed for the selection of differentially expressed genes. Additionally, WGCNA, machine learning, and Genetic Perturbation Similarity Analysis (GPSA) were utilized to identify key genes associated with NAC treatment response. ConsensusClusterPlus was employed for unsupervised clustering. Rt-qPCR and WB were conducted to assess gene expression and protein levels in clinical tissues and cell lines. The Seahorse XF96 Extracellular Flux Analyzer was utilized to evaluate Extracellular Acidification Rate (ECAR) and Oxygen Consumption Rate (OCR). The "pRRophetic" package was used for drug sensitivity prediction, while CB-Dock2 was applied for molecular docking and optimal pose presentation. Spatial transcriptomic analysis was based on the CROST database.

**Results:** Eleven biomarkers were identified associated with NAC treatment response in BC patients, with FOXA1 identified as a pivotal hub gene among them. The expression levels of FOXA1 showed a significant positive correlation with genomic stability and a marked negative correlation with the homologous recombination deficiency (HRD) score. Downregulation of the FOXA1 gene resulted in reduced glycolysis in MCF-7 cells.Additionally, FOXA1 were found to serve as a biomarker for both NAC and PARP inhibitor treatment sensitivity in BC patients. Spatial transcriptomic analysis indicates significantly elevated infiltration of T follicular helper (T-FH) cells and mast cells surrounding tumors exhibiting high FOXA1 expression.

**Conclusion:** In summary, our study involved the analysis of diverse sequencing datasets derived from various FNA samples to identify biomarkers sensitive to NAC, thereby offering novel insights into resources for future personalized clinical treatment strategies.

## Introduction

Among all women in the world, breast cancer (BC) has the highest incidence rate and the second highest case fatality rate; Therefore, it seriously damages the physical and mental health of women, bringing a heavy social and family medical burden^1^. The management of BC encompasses a multidisciplinary approach, primarily consisting of surgical intervention, chemotherapy, radiotherapy, endocrine therapy, and targeted therapy. Among these, the most frequently performed surgical modality is the modified radical mastectomy for BC^2^. However, when the tumor is large or has extensive axillary lymph node metastasis, it is considered impossible to perform a one-stage surgical resection. Preoperative neoadjuvant chemotherapy (NAC) can reduce the size of the tumor and increase the possibility of radical surgery^3^. Due to the heterogeneity of tumors, some patients with BC have a high response to NAC, with significant tumor regression and a relatively lower risk of postoperative recurrence^4^. However, other patients who are not sensitive to NAC may face difficulties in undergoing radical surgery and a high risk of postoperative recurrence. Therefore, screening and identifying relevant biomarkers for NAC treatment responsiveness, and predicting the responsiveness of BC patients to NAC treatment through these biomarkers, have high clinical application value.

Fine needle aspiration (FNA) biopsy is one of the most commonly used methods for evaluating the nature of nodules in the breast^5^. FNA technology is fast and simple, and has great speed advantages and cost-effectiveness in BC diagnosis^6^. FNA combined with high-throughput sequencing technology is a more convenient way for clinicians to assess tumor heterogeneity between different BCs before developing treatment plans for BC tumors, in order to develop personalized treatment plans. However, there are still few studies on using the BC gene expression profile obtained by this FNA to predict the response to NAC treatment. Therefore, further exploration and development of new molecular typing methods for BC tumors based on FNA to prospectively predict the response to NAC treatment are beneficial for the formulation of appropriate treatment plans.

In summary, this study aims to extensively collect sequencing datasets of BC tumors obtained through FNA prior to the initiation of NAC treatment. The objective is to explore the heterogeneity between tumors of patients with responsive and non-responsive outcomes to NAC, and to develop novel molecular classification schemes to optimize the prediction of NAC treatment response. Furthermore, we delved deeper into the identification of key genes related to NAC treatment responsiveness and their potential biological functions, which was verified through experimental methods. The flowchart of this study is shown in Supplementary Fig. 1.

## Materials and Methods

### Acquisition of data

Gene Expression Omnibus (GEO; http://www.ncbi.nlm.nih.gov/geo/) was used to obtain the gene expression profile and clinical information^7^, ^8^. For this study, the following inclusion and exclusion criteria were used to acquire appropriate datasets. (1) The sample size cannot be too small (n>50). (2) Patients in datasets had received standardized NAC regimen prior to surgery. (3) The expression profile data was derived from specimens obtained through FNA before NAC. The study ultimately included five data sets, including GSE20194 (n=278), GSE20271 (n=178), GSE22093 (n=103), GSE23988 (n=61) and GSE42822 (n=91). Supplementary Table 1 summarized the clinical information of patients in these datasets. The “InSilicoMerging” R package was used to merge the above datasets into “merge data”, using “COMBAT” for batch effect removing^9^. The principle of combat removing batch effects is mainly based on the adjustment of location and scale, L/S. This method is of great significance in improving data quality, enhancing data consistency, ensuring the accuracy and reliability of analysis results, improving research reproducibility, and promoting data integration^10^, ^11^.

### Weighted Gene Co-Expression Network Analysis (WGCNA)

After removing the top 50% genes with the smallest Median Absolute Deviation (MAD) and using the "goodSamplesGenes" algorithm to remove outliers, the remaining samples and genes were included in the WGCNA analysis^12^. We set the threshold as follows: minimum module size = 30; sensitivity = 3; module merging threshold = 0.25; β=7^13^. Finally, we used different colors of modules to represent different clusters of co-expressed genes, and the set of genes that were deemed unable to be assigned to any module was grouped into the “grey” module. Then, analysis was conducted on the correlation between module eigengenes and clinical traits, aiming to identify notable modules that exhibited a significant association with clinical characteristics. By setting the threshold to Module Membership (MM)>0.8 and Gene Significance (GS)>0.1, we obtained the hub genes in each co-expression module^8^, ^13^-^16^.

### Differential analysis and Gene set enrichment analysis

The gene differential analysis was performed using the “LIMMA” package, with a threshold set at an absolute fold change greater than 1.5 and a False Discovery Rate (FDR) less than 0.05^17^. Gene enrichment analysis was completed through the Metascape database (https://metascape.org/gp/index.html#/main/step1), which integrates all KEGG Pathways, GO Biological Processes, Reactome Gene Sets, Canonical Pathways, CORUM, and WikiPathways^18^. In addition, we leveraged the single-sample Gene Set Enrichment Analysis (ssGSEA) approach to derive enrichment scores for specific pathways across different samples, thereby evaluating the activation status of these pathways^19^.

### Unsupervised consensus clustering

The process of cluster analysis was executed utilizing the ConsensusClusterPlus package, employing an agglomerative pam clustering methodology with 1-Pearson correlation distances^20^. Additionally, the analysis involved resampling 80% of the samples and iterating this procedure 10 times. The identification of the optimal cluster count was achieved by evaluating the empirical cumulative distribution function plot.

### Genetic Perturbation Similarity Analysis (GPSA)

The GPSA database (https://www.gpsadb.com/) has curated 3048 gene perturbation RNA-seq datasets and performed differential analysis on each of them, resulting in 6096 gene sets that correspond to specific gene perturbations such as gene knockouts^21^. The gene perturbation RNA-seq datasets in the GPSA database primarily originate from cell lines. These gene sets provide insights into the transcriptional changes that occur when specific genes are perturbed, enabling researchers to gain a deeper understanding of the functional roles and interactions of genes within biological systems. When the pattern of differentially expressed genes obtained through differential analysis based on a certain phenotype grouping is similar to that of corresponding gene sets derived from gene perturbations, it suggests a close association between that phenotype and the gene perturbations that led to the generation of those gene sets. This indicates that the phenotype may be influenced or mediated by the functional changes caused by the perturbations of the specific genes within those gene sets.

### Machine learning

There are three machine learning methods included in this study, including Support Vector Machine (SVM), Least Absolute Shrinkage and Selection Operator (LASSO), and XGboost^22^-^24^. SVM is implemented using the "e1071" R package, and all parameters of SVM are set to default values^25^. The “glmnet” package was utilized to conduct Lasso modeling, incorporating a 10-fold cross-validation approach^26^. The Lasso parameter Alpha was set to 0.1^27^. We utilize the R package “xgboost” for training XGBoost models as well as for estimating SHapley Additive exPlanations (SHAP) values, where SHAP values exceeding 30 are considered as indicating key genes^28^. All other settings in the XGBoost analysis are set to their default values.

### Pan-cancer analysis

The expression data for Pan-Cancer, as well as the comprehensive TCGA PanCancer Atlas dataset, were sourced from UCSC Xena (accessible at http://xena.ucsc.edu/). To illustrate the gene expression levels, box plots were employed. Furthermore, the normalized gene expression values, measured in transcripts per million (TPM), are presented in log2(TPM + 1) format for each individual gene.

### Reverse Transcription-Quantitative PCR (RTqPCR) and Immunohistochemistry (IHC)

RNA extraction was performed utilizing TRIzol reagent sourced from Ambion, USA. Subsequently, the conversion of mRNA to cDNA was achieved through the application of PrimeScriptTM RT Master Mix from Takara, Japan. Quantification of gene transcripts was conducted via the RT-qPCR assay, employing ChamQ SYBR qPCR Master Mix provided by Vazyme, China. Evaluation of relative gene expression levels was conducted using the 2-ΔΔCT method, with GAPDH serving as the internal reference gene. For the measurement of FOXA1 and GAPDH expression levels, specific primer sequences were utilized: FOXA1's forward primer was 5′-GGAGGAGCGGATTCAGGAGGAG-3′ and its reverse primer was 5′-AGCAGATGATGTTGGCGGTAATGG-3′; GAPDH's forward primer was 5′-GGAGCGAGATCCCTCCAAAAT-3′ and its reverse primer was 5′-GGCTGTTGTCATACTTCTCATGG-3′. To ensure accuracy, the experiment was replicated thrice and the average values were calculated. The RT-qPCR method was utilized to determine gene expression levels.

The study involved samples obtained from eight BC patients admitted to The Third Affiliated Hospital of Anhui Medical University. These samples were subjected to RT-qPCR analysis. Prior to inclusion in the study, all patients provided informed consent.

For IHC analysis, data was retrieved from the HPA database and The Third Affiliated Hospital of Anhui Medical University. The Average Optical Density (AOD) was adopted as the scoring method for statistical analysis. Professional pathologists utilized the ImageJ software to measure the AOD, with a minimum of three measurements per sample taken to determine the mean AOD values.

### Western blotting analysis

Following the digestion process utilizing RIPA, the total protein contents of both MCF-7 cells were successfully extracted. To quantify the cellular protein levels, we employed a BCA kit from Beyotime (P0011). For protein separation, a 10% SDS-PAGE gel was utilized, with each lane loaded with 50 grams of protein. The isolated proteins were then transferred onto polyvinylidene difluoride (PVDF) membranes, which were further blocked with 5% milk to minimize non-specific binding. For primary antibody detection, we selected rabbit-derived anti-FOXA1 (Abcam, cat. no. ab170933, dilution 1:1,000), anti-GLUT1 (Abcam, cat. no. ab115730, dilution 1:100,000), anti-HK2 (Abcam, cat. no. ab209847, dilution 1:1,000), anti-LDHA (Abcam, cat. no. ab52488, dilution 1:3,000), anti-GAPDH (Abcam, cat. no. ab8245, dilution 1:3,000) and anti-β-Actin (Abcam, cat. no. ab8227, dilution 1:1,000). Subsequently, following thorough washing with TBS-T buffer, the membranes underwent a 1-hour incubation period at 4°C with secondary antibodies conjugated to horseradish peroxidase (HRP), sourced from Abcam (cat. no. ab6721, dilution 1:2,000). In order to achieve color development, we employed the enhanced chemiluminescence (ECL) chemical hypersensitivity chromogenic reagent kit. Finally, the intensity of the proteins was precisely measured using ImageJ software.

### Cell culture

We procured the human BC cell line MCF-7 from the American Type Culture Collection (ATCC). These cells were cultured in Dulbecco's Modified Eagle's Medium (DMEM; Gibco, Massachusetts, USA; catalog number: 31966047) at 37 °C and 5% CO₂, supplemented with 10% fetal bovine serum (FBS; Gibco, Massachusetts, USA; catalog number: 10500064) and 1% penicillin-streptomycin (Gibco, Massachusetts, USA; catalog number: 15070063). To ensure cellular stability, all experiments were conducted using cells that had undergone fewer than 25 passages. FOXA1 expression in MCF-7 cells was knocked down through transfection with FOXA1-specific small interfering RNA (siFOXA1; GenePharma, Shanghai, China), facilitated by Lipofectamine 3000 (Invitrogen, Carlsbad, CA, USA), and executed in accordance with the manufacturer's prescribed protocol. The siRNA sequences were precisely designed as: 5′-GGACUUCAAGGCAUACGAATT′ (sense) and 5′-UUCGUAUGCCUUGAAGUCCAG-3′ (antisense). Non-targeting siRNA (siNC) served as the control in this experimental setup.

### Assessment of cellular metabolic activity

To evaluate Extracellular Acidification Rate (ECAR) and Oxygen Consumption Rate (OCR), Seahorse XF96 Extracellular Flux Analyzer was implemented, referencing a prior study^29^, ^30^. In summary, 4 × 10^4 cells were plated ontoXF96 cell culture microplates. The Seahorse buffer was composed of DMEM, phenol red, 25 mM glucose, 2 mM sodium pyruvate, and 2 mM glutamine. For ECAR assessment, 10 mM glucose, 1 μM oligomycin, and 100 mM 2-deoxy-glucose (2-DG) were sequentially injected to quantify ECAR values^31^. Subsequently, after baseline respiration monitoring, 1 μM oligomycin, 1 μM FCCP (Carbonyl cyanide-4-(trifluoromethoxy) phenylhydrazone), and 1 μM rotenone were automatically added to the XF96 cell culture microplates for OCR measurement^32^ .

### Predicting drug sensitivity and molecular docking

To determine the half maximal inhibitory concentration (IC50), a regression analysis was conducted using the pRRophetic package in the R programming environment. The "pRRophetic" package, which we utilized for our analysis, was developed based on the Genomics of Drug Sensitivity in Cancer (GDSC) database^33^. Our primary focus was on predicting the IC50 values for PARP inhibitors, specifically Rucaparib (AG-014699), Veliparib (ABT-888), and Olaparib (AZD2281). Molecular docking was performed using the CB-Dock2 online tool (https://cadd.labshare.cn/cb-dock2), which is a molecular docking program based on AutoDock Vina^34^. All parameters were set to their default values. The molecular structures of the drugs were obtained from the PubChem database (https://pubchem.ncbi.nlm.nih.gov/), while the protein structure of FOXA1 was sourced from the RCSB Protein Data Bank (https://www.rcsb.org/)^35^, ^36^.

### Statistical analyses

R software, version 4.0.4, was employed for the execution of all statistical procedures. To compare continuous variables, the Wilcoxon/Kruskal-Wallis Test was adopted^37^. For evaluating differences in proportions, the Chi-Square test was utilized. Statistical significance was defined by a p-value below 0.05. Furthermore, a receiver operating characteristic (ROC) curve was plotted to evaluate the predictive efficacy of the prognostic prediction model. For correlation analysis, the Spearman correlation method was implemented.

## Results

### Identification of key modules and biomarkers of NAC for BC through WGCNA method

**Figure 1:**
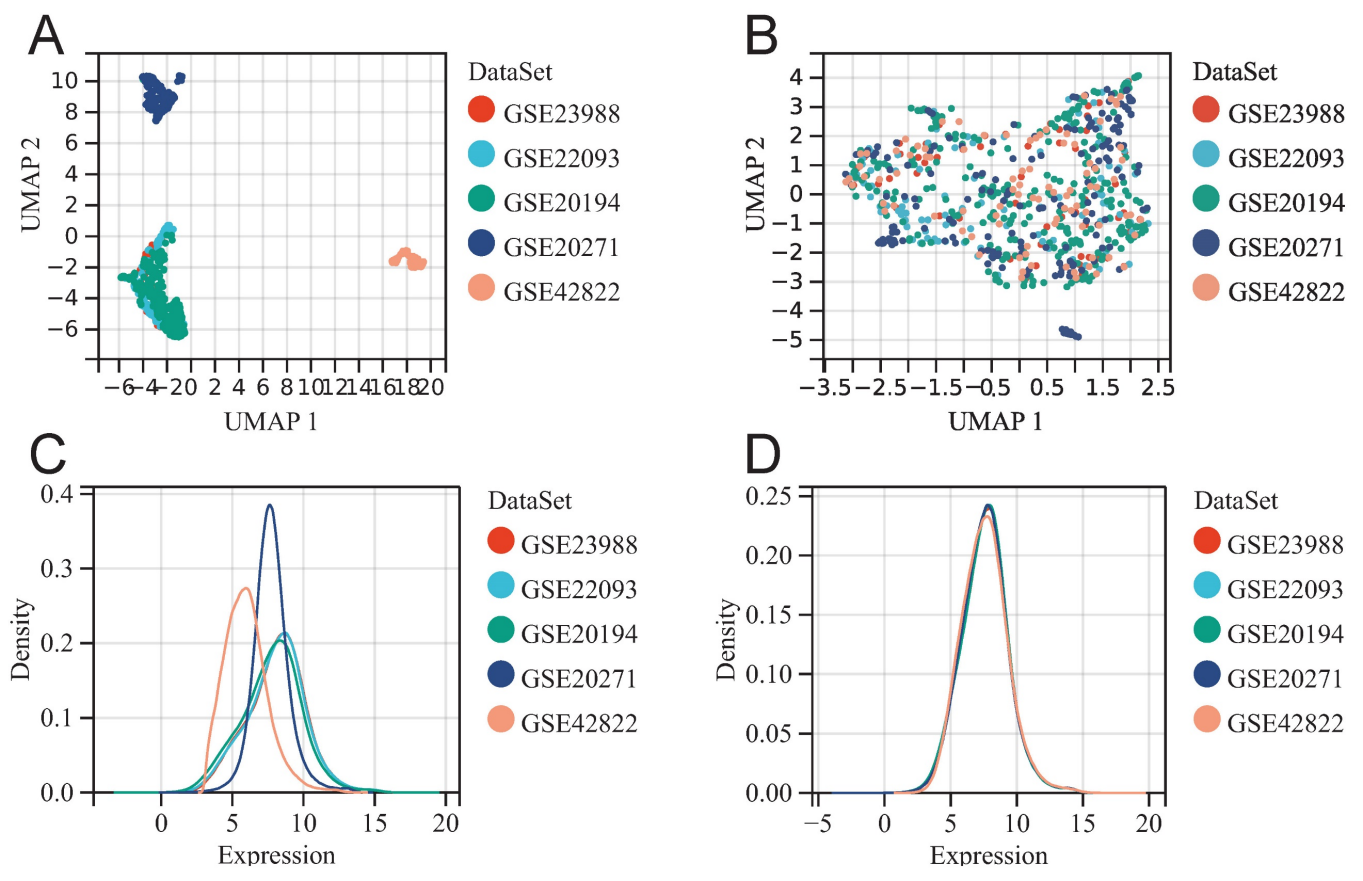
Merging datasets and removing batch effect. UMAP diagram before (A) and after (B) batch effect removal. The density plot before (C) and after (D) batch effect removal.

Before batch effect removal, UMAP plot shows distinct clustering for each dataset (Fig. 1A), indicating batch effects. Post-removal, samples from various datasets cluster interchangeably (Fig. 1B), suggesting effective elimination. Density plot reveals significant differences in sample distribution before removal (Fig. 1C), suggesting batch effects. Post-removal, distributions converge with similar means and variances (Fig. 1D). The above results suggested that we successfully removed batch effects when merging GSE20194, GSE20271, GSE22093, GSE23988, and GSE42822.

**Figure 2:**
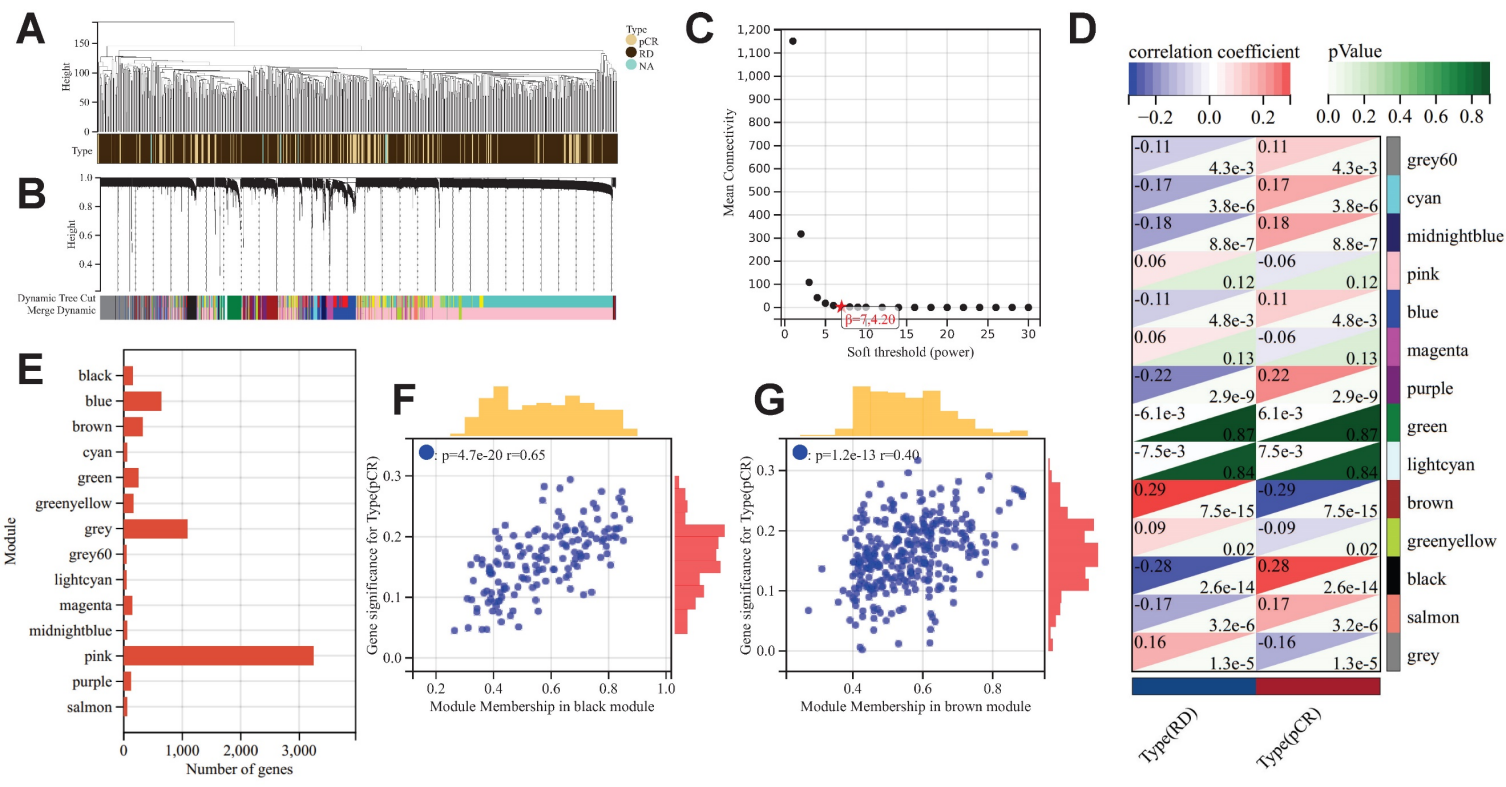
The results of WGCNA. (A) Clustering samples for merge data. (B) Cluster dendrogram indicating co-expressed gene modules. (C) Identification of soft threshold 9for WGCNA. (D) WGCNA and module-trait correlation analysis. (E) The number of genes in each gene module. Scatter plot of correlation between GS and MM for black module (F) and brown module (G).

After excluding outlier samples and genes, we constructed a scale-free co-expression network with a β value set to 7 (Fig. 2A-2C). A total of 14 co-expression gene modules were identified, and the genes within each module are compiled in Supplementary Table 2 (Fig. 2D). The black module showed the most significant positive correlation with pCR after NAC treatment in BC patients (R=0.28, *p*<0.001), while the brown module exhibited the most significant negative correlation (R=-0.29, *p*<0.001). Consequently, the genes within these two modules were identified as "NAC-WGCNA-related genes" and were included in the subsequent analysis. The black module contains a total of 153 genes, while the brown module comprises 324 genes (Fig. 2E). Upon correlating the modules with clinical characteristics, the MM and GS values for the black and brown modules were determined (Fig. 2F&2G). Utilizing the threshold values of |MM| greater than 0.8 and |GS| greater than 0.1 as standards for filtering core genes (NAC-WGCNA-Hub genes), a total of 14 hub genes were identified within the black module, while 10 hub genes were selected from the brown module (Supplementary Table 3).

**Figure 3:**
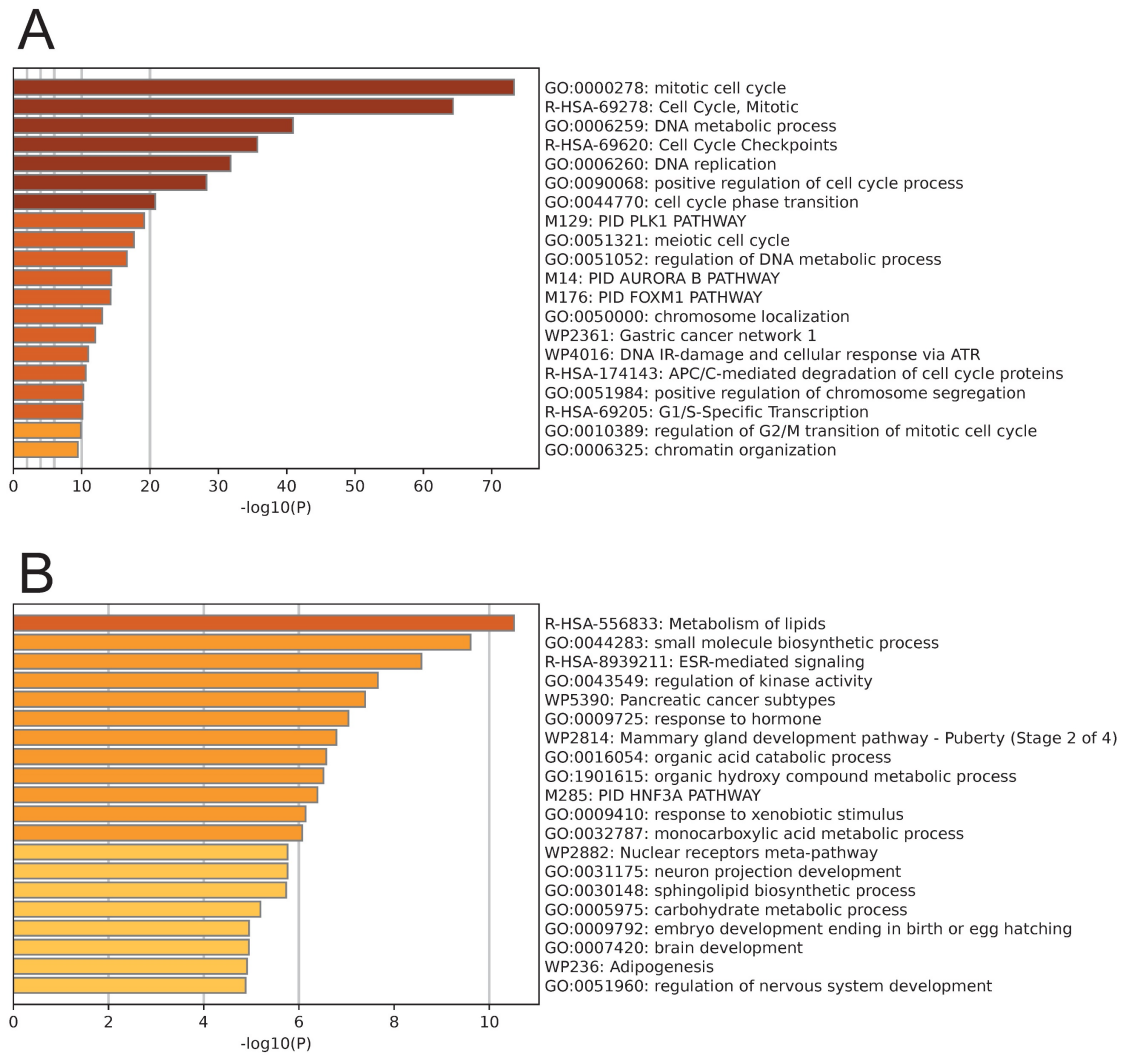
Enrichment analysis results of genes in the black module (A) and brown module (B).

Pathway enrichment analysis was performed on the genes from the black module and the brown module. The genes in the black module were primarily enriched in pathways related to the cell cycle, such as the mitotic cell cycle, DNA metabolic process, DNA replication, and positive regulation of the cell cycle process (Fig. 3A). Conversely, the genes in the brown module were primarily enriched in cell metabolism-related pathways, including metabolism of lipids, small molecule biosynthetic process, regulation of kinase activity, organic acid catabolic process, and organic hydroxy compound metabolic process (Fig. 3B).

### Identification of DEGs between BC patients with different treatment response of NAC

**Figure 4:**
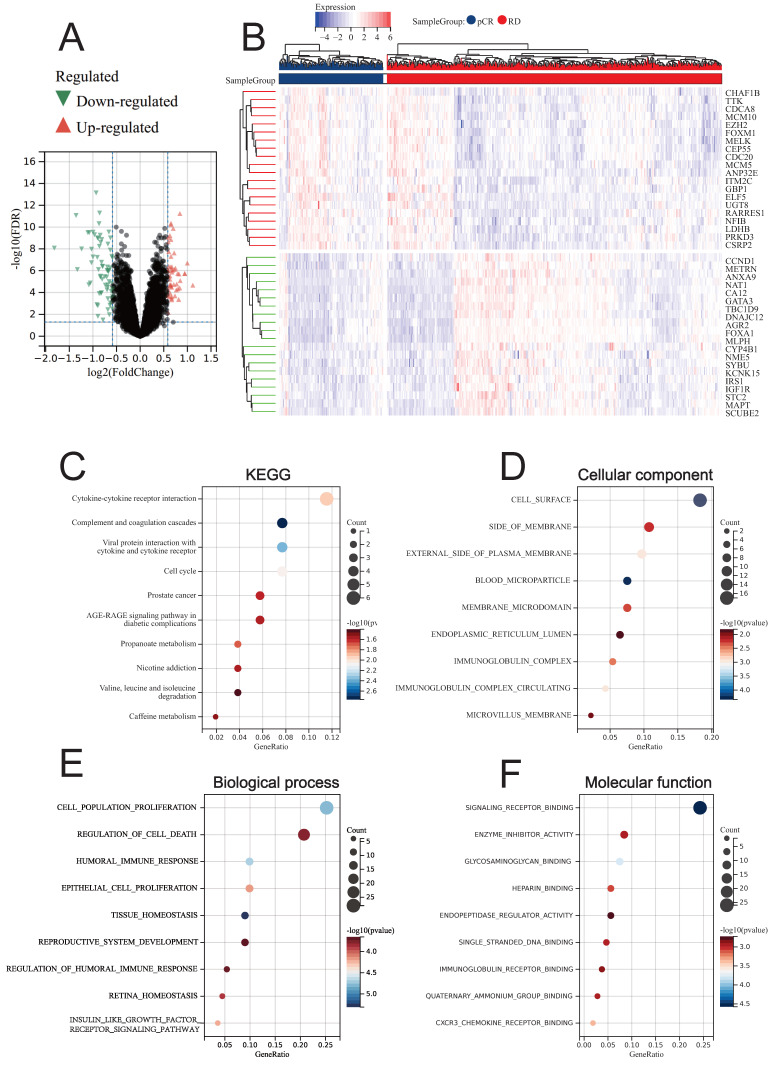
Differential analysis results between pCR and RD patients. (A) Volcano plot showing DEGs. (B) Heatmap showing the top 20 upregulated and downregulated genes in pCR patients. (C) KEGG enrichment analysis of DEGs. GO enrichment analysis for DEGs, including CC (D), BP (E), and MF (F).

Through LIMMA, we identified DEGs between BC patients who achieved pCR and RD after NAC treatment (NAC-DEGs), and found that a total of 69 genes were down-regulated in the pCR group and 52 genes were up-regulated in the pCR group (Fig. 4A). The top 20 genes that were up-regulated and down-regulated are displayed in the heatmap (Fig. 4B). KEGG enrichment analysis revealed that NAC-DEGs were primarily enriched in Cytokine-cytokine receptor interaction, Complement and coagulation cascades, and Viral protein interaction with cytokine and cytokine receptor (Fig. 4C). GO enrichment analysis revealed that NAC-DEGs were primarily enriched in cell surface, side of membrane, cell population proliferation, regulation of cell death, and signaling receptor binding (Fig. 4D-4F).

### Single Nucleotide Variation (SNV) and Copy Number Variation (CNV) landscape of key genes related to NAC treatment response

**Figure 5:**
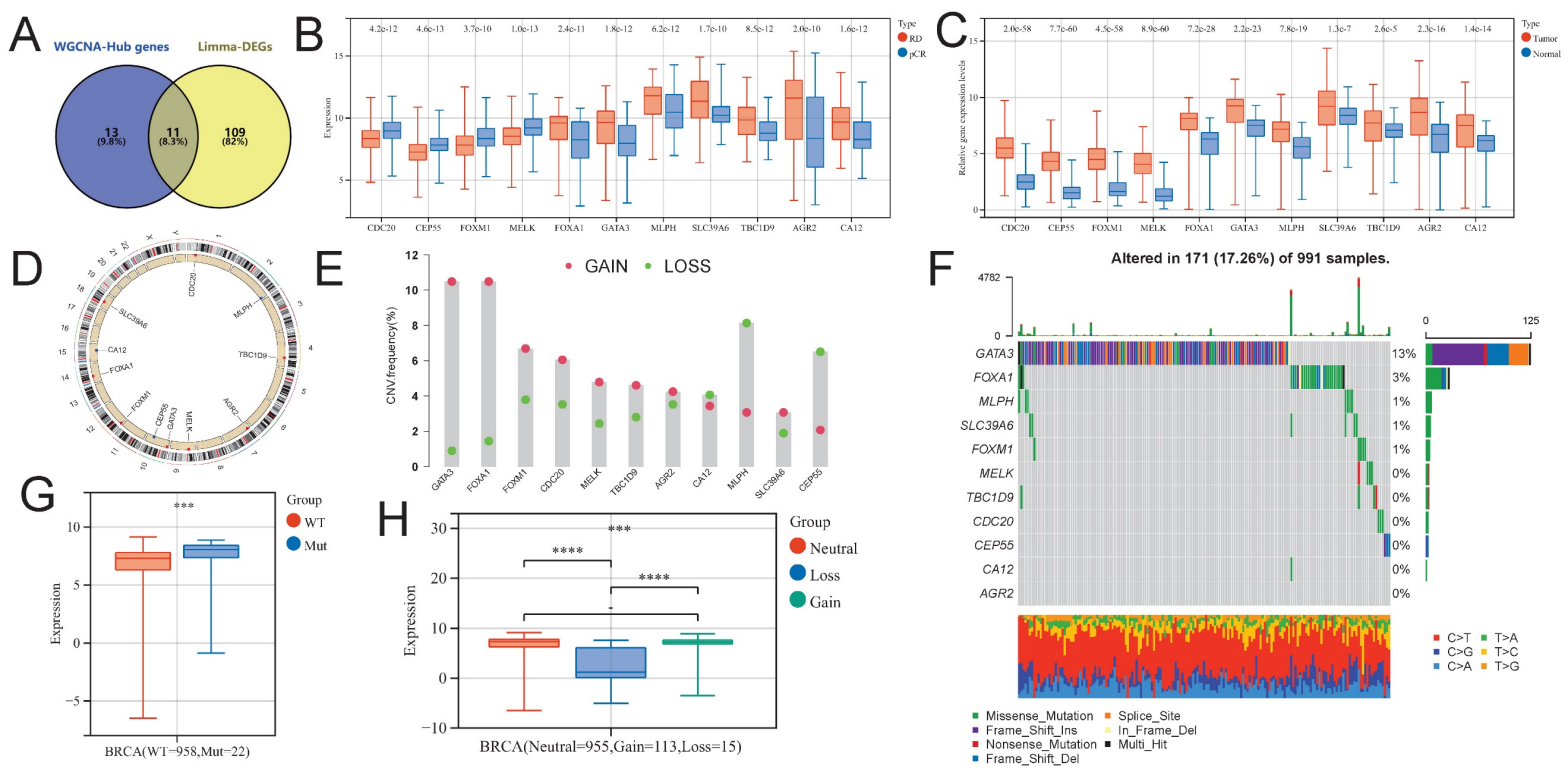
Identification of NAC-pCR related genes. (A) The Venn diagram showing the intersection genes of the Hub genes in the black and brown modules in WGCNA, and the DEGs obtained from limma. (B) The expression level differences of NAC-pCR related genes between pCR and RD patients. (C) The expression levels of NAC-pCR related genes in BC tumor and normal breast tissue samples. (D) The chromosomal location of NAC-pCR related genes. (E) Gain and loss frequencies of CNVs of NAC-pCR related genes in TCGA-BRCA's BC patients. (F) Waterfall plots of mutant NAC-pCR related genes. Transcriptomic expression levels of FOXA1 with different SNV (G) and CNV statuses (H).

Using the venny2.1.0 tool (https://bioinfogp.cnb.csic.es/tools/venny/index.html), we intersected NAC-DEGs and NAC-DEGs, and found a total of 11 intersecting genes (NAC-pCR related genes; Fig. 5A). Four of the 11 genes (CDC20, CEP55, FOXM1 and MELK) had lower expression levels in BC patients with pCR, while the remaining genes were up-regulated in BC patients with pCR (Fig. 5B). In addition, all genes showed significantly up-regulated expression levels in BC tumor tissues (Fig. 5C). We further explored the SNV and CNV landscape of these 11 genes in the TCGA-BRCA dataset. The chromosomal locations of all genes are displayed in Figure 5D. In BC tumors, more than 10% of samples have CNV gain for GATA3 and FOXA1, while the highest proportion of CNV loss for MLPH is about 8% in BC tumor samples (Fig. 5E). Among the 991 BC tumor samples, GATA3 had the highest frequency of somatic SNVs, accounting for 13%; followed by FOXA1, accounting for 3%; AGR2, CA12, CEP55, CDC20, TBC1D9, and MELK had very few somatic SNVs (Fig. 5F). We further explored the impact of SNV and CNV on the expression level of FOXA1 gene, and found that the expression level of FOXA1 gene was significantly up-regulated after SNV mutation, while the expression level of FOXA1 gene was significantly down-regulated after CNV deletion (Fig. 5G).

### Construction of a new molecular typing scheme to predict the response of BC patients to NAC treatment

**Figure 6:**
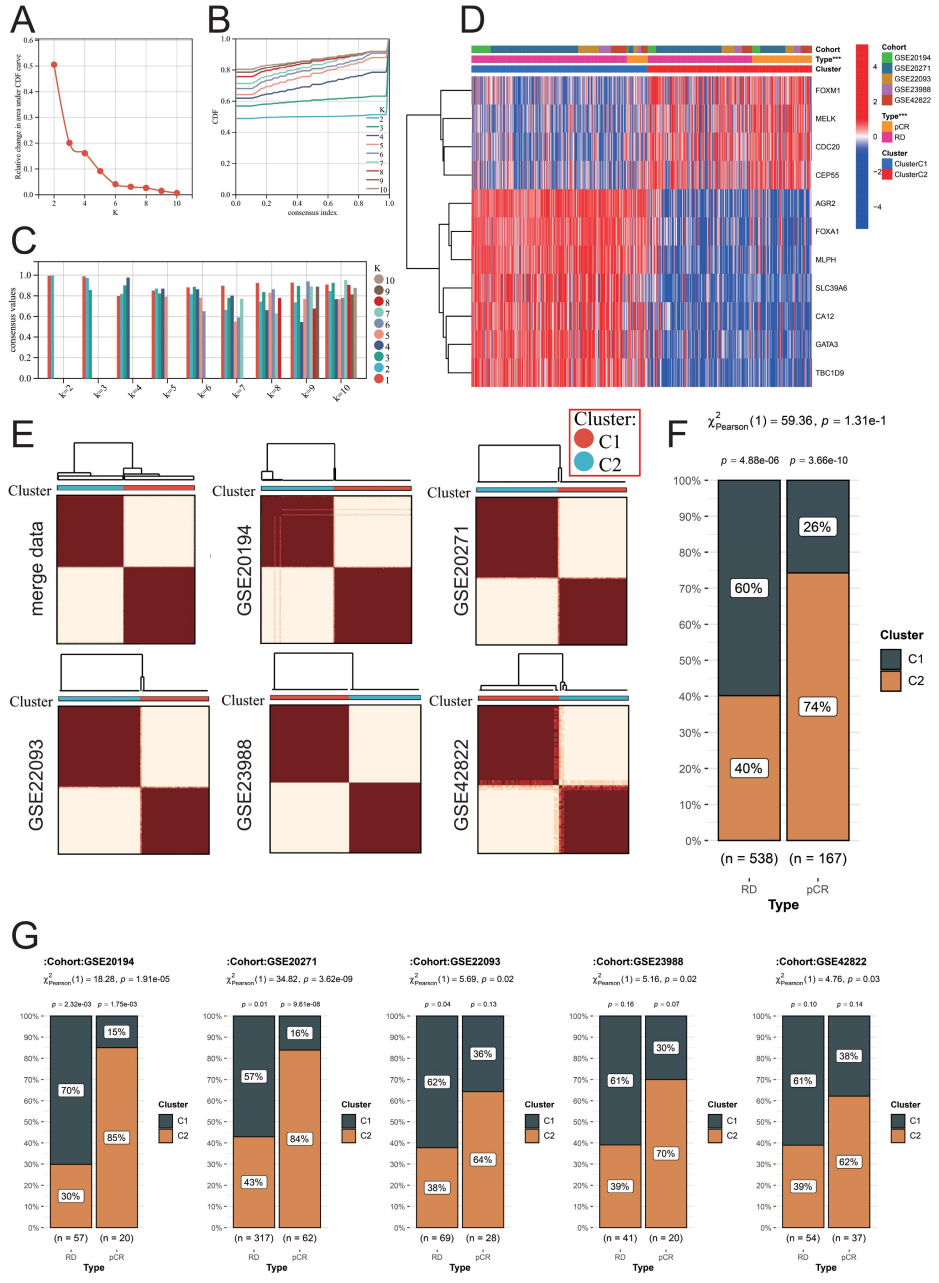
Identification of a new molecular classification scheme. (A) Variation of area under the CDF curve. (B) CDF curves when k=2-10. (C) A visualization of cluster-consensus trends, depicting the average pairwise consensus score for each subtype across varying k-values. (D) Heatmap of expression levels of NAC-pCR related genes among different clusters. (E) Heatmap of unsupervised consensus clustering. In the merged data (F) and individual datasets (G), the proportion of patients in different clusters among RD and pCR BC patients.

We used the above 11 NAC-pCR related genes for unsupervised clustering to construct a new molecular typing scheme that can predict the therapeutic response of BC patients to NAC based on the expression profiles of samples obtained from FNA. According to the area under the CDF curve and the intra-group consistency evaluation, the optimal number of clusters in the merge data is K=2 (Fig. 6A-6C). Therefore, all the samples obtained through FNA were classified into two clusters. CDC20, CEP55, FOXM1, and MELK are highly expressed in cluster 2 (C2), while the remaining genes are highly expressed in cluster 1 (C1) (Fig. 6D). Not only are the samples in the merge data well divided into two clusters, but the respective independent datasets GSE20194, GSE20271, GSE22093, GSE23988, and GSE42822 can also be well divided into two clusters using this molecular typing scheme (Fig. 6E). This molecular typing scheme has a good predictive ability for the response of BC patients to NAC. Patients classified into cluster 2 predominantly constitute the majority of patients who achieve pCR, and this phenomenon is not only observed in the merge data but also consistent across the independent datasets (Fig. 6F-6G).

Moreover, the landscape of NAC-pCR related genes obtained through FNA can also effectively diagnose BC tumors. Through three dimensionality reduction methods (PCA, UMAP, tSNE) using 11 NAC-pCR related genes, tumor samples and normal samples can be effectively separated. Through three dimensionality reduction methods (PCA, UMAP, tSNE) using 11 NAC-pCR related genes, tumor samples and normal samples can be effectively separated (Supplementary Fig. 2A). Furthermore, individual NAC-pCR related genes also demonstrate diagnostic efficacy for BC tumors, particularly CDC20, CEP55, FOXM1, and MELK, with AUC values exceeding 0.9 (Supplementary Fig. 2B).

### Differences in pathways between BCs with different therapeutic responses to NAC

We used the ssGAEA algorithm to evaluate the enrichment of 22 tumor-related pathways in different groups of BC patients, and the key genes included in each pathway were summarized in Supplementary Table 4. Significant upregulation was observed in the cell DNA damage repair and cell cycle-related pathways in BC patients who achieved pCR. Additionally, immune-related antigen presentation and processing, as well as CD8+ T cell function-related pathways, also exhibited significant upregulation (Supplementary Fig. 3A). The extensive differences in pathway enrichment between cluster C1 and cluster C2, which were classified using the our molecular typing scheme, suggest that this molecular typing approach effectively distinguishes BC tumors with different heterogeneities (Supplementary Fig. 3B). It is noteworthy that the four genes (CDC20, CEP55, FOXM1 and MELK) that were up-regulated in BC patients who achieved pCR showed a significant positive correlation with pathways related to DNA damage repair and cell cycle (Supplementary Fig. 3C). This is indeed consistent with the fact that some drugs in the NAC regimen target DNA replication during the cell cycle.

### Machine learning and GPSA analysis identify FOXA1 as a key gene for NAC treatment response

**Figure 7:**
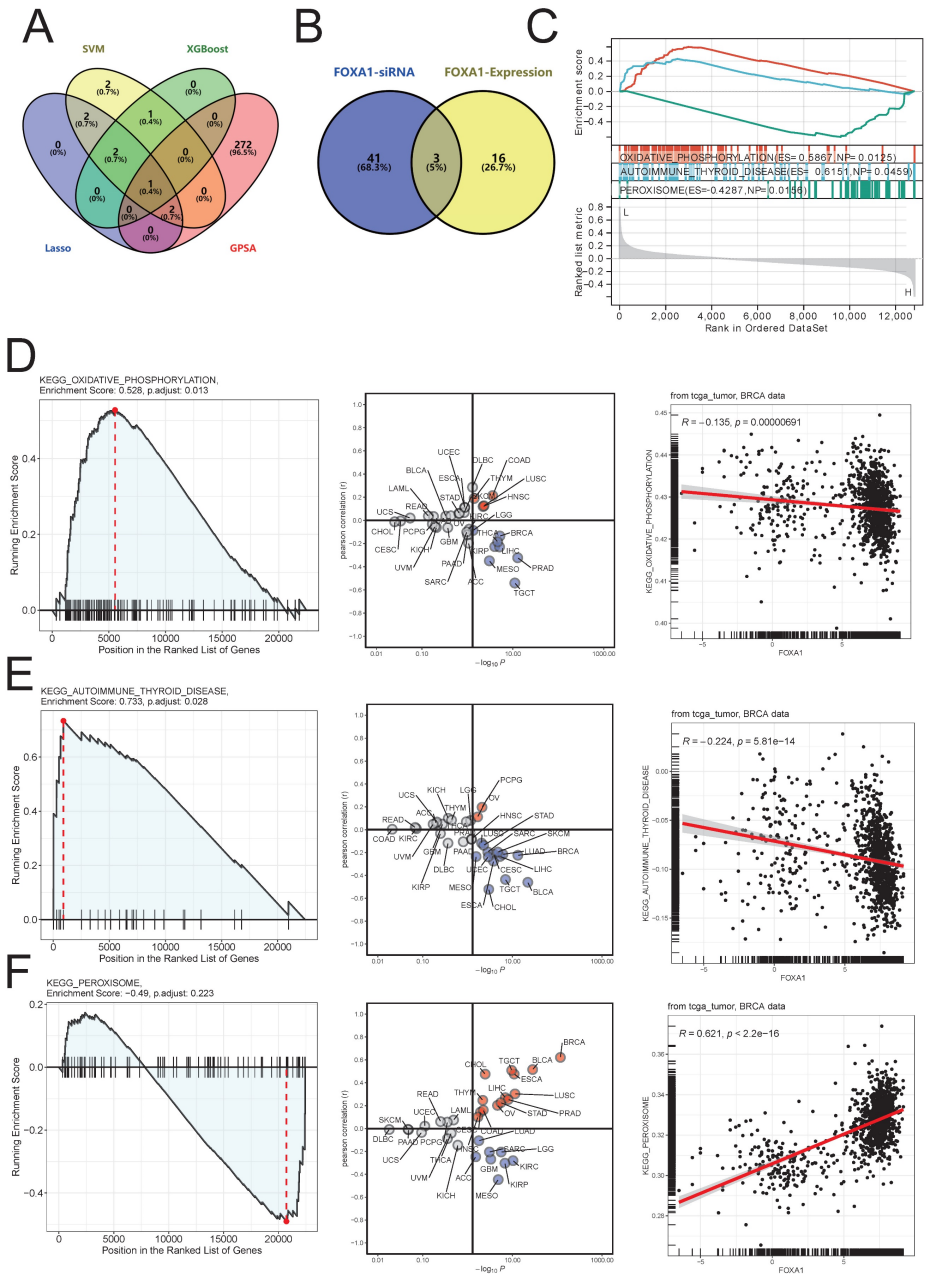
Identification of key genes associated with pCR of NAC. (A) Intersection genes obtained by three machine learning methods and GPSA analysis. (B) Venn diagram of the intersection between significantly altered KEGG pathways after FOXA1 knockdown and KEGG pathways significantly associated with FOXA1 expression levels as indicated by GSEA. (C) GSEA analysis results of three intersecting KEGG pathways with changes in FOXA1 expression. (D) The OXIDATIVE PHOSPHORYLATION pathway is significantly upregulated in the FOXA1 knockdown group (left). Pan-cancer analysis of the correlation between the OXIDATIVE PHOSPHORYLATION pathway and FOXA1 expression levels (middle). In TCGA-BRCA, the OXIDATIVE PHOSPHORYLATION pathway and FOXA1 expression levels show a significant negative correlation (right). (E) The AUTOIMMUNE THYROID DISEASE pathway is significantly upregulated in the FOXA1 knockdown group (left). Pan-cancer analysis of the correlation between the AUTOIMMUNE THYROID DISEASE pathway and FOXA1 expression levels (middle). In TCGA-BRCA, the AUTOIMMUNE THYROID DISEASE pathway and FOXA1 expression levels show a significant negative correlation (right). F) The PEROXISOME pathway is significantly downregulated in the FOXA1 knockdown group (left). Pan-cancer analysis of the correlation between the PEROXISOME pathway and FOXA1 expression levels (middle). In TCGA-BRCA, the PEROXISOME pathway and FOXA1 expression levels show a significant positive correlation (right).

We further used three machine learning methods, namely SVM (Supplementary Fig. 4A), XGBoost (Supplementary Fig. 4B), and Lasso (Supplementary Fig. 4C), as well as the GPSA algorithm to screen key genes among the 11 NAC-pCR related genes. The GPSA algorithm identified a total of 275 gene perturbations related to achieving pCR with NAC treatment (Supplementary Table 5). By integrating machine learning methods and GPSA, FOXA1 was identified as a key gene associated with pCR (Fig. 7A). In the D21558 dataset in GPSA (Supplementary Table 5), the gene expression pattern that was up-regulated after knockdown of FOXA1 was inversely correlated with BC patients who achieved pCR; the gene expression pattern that was down-regulated after knockdown of FOXA1 was positively correlated with BC patients who achieved pCR (Supplementary Fig. 4D,4E). Moreover, after knocking down FOXA1, the variation of Hallmarks pathway in cells and the variation of Hallmarks pathway in BC patient samples that reached pCR showed a strong significant negative correlation R = -0.971, p < 0.001 (Supplementary Fig. 4F).

Besides FOXA1, other genes also identified by GPSA as having undergone gene perturbation, such as gene knockouts, exhibit gene expression patterns similar to or opposite to those of BCs that have reached pCR. We showed the top 20 genes with the highest GPSI scores and the changes in the enrichment of 50 Hallmarks pathways after perturbation of these genes (Supplementary Fig. 5A,5B). GPSI is a measure of the variation in gene expression landscape and the similarity of inputted preset differential genes and rank ordering before and after gene perturbation. A higher GPSI score indicates a greater correlation. The percentage of times each hallmark gene set was enriched was exhibited in Supplementary Fig. 5C.

### The potential biological function of FOXA1 in BC tumors

In the GEPIA database, FOXA1 is significantly overexpressed in BC tumor tissues, and the FOXA1 protein level in BC tumor sample tissues in the HPA database is also significantly higher than that in normal tissues (Supplementary Fig. 6A,6B). FOXA1 is located in the cytoplasm of many tumor cell lines, not only in the breast cancer cell line MCF-7 (Supplementary Fig. 6C). In addition, we used the breast cancer spatial transcriptome data project number: VISDS000554 in the CROST database (https://ngdc.cncb.ac.cn/crost/home) to explore the expression pattern of FOXA1 (Supplementary Fig. 6D). FOXA1 is mainly highly expressed in tumor sites; compared to other immune cells, T follicular helper T-FH cells and Mast Cells have significantly higher levels of infiltration around tumors with high FOXA1 expression. This suggests that the expression of FOXA1 may have a potential impact on the pattern of immune cells infiltrating within tumors.

**Figure 8:**
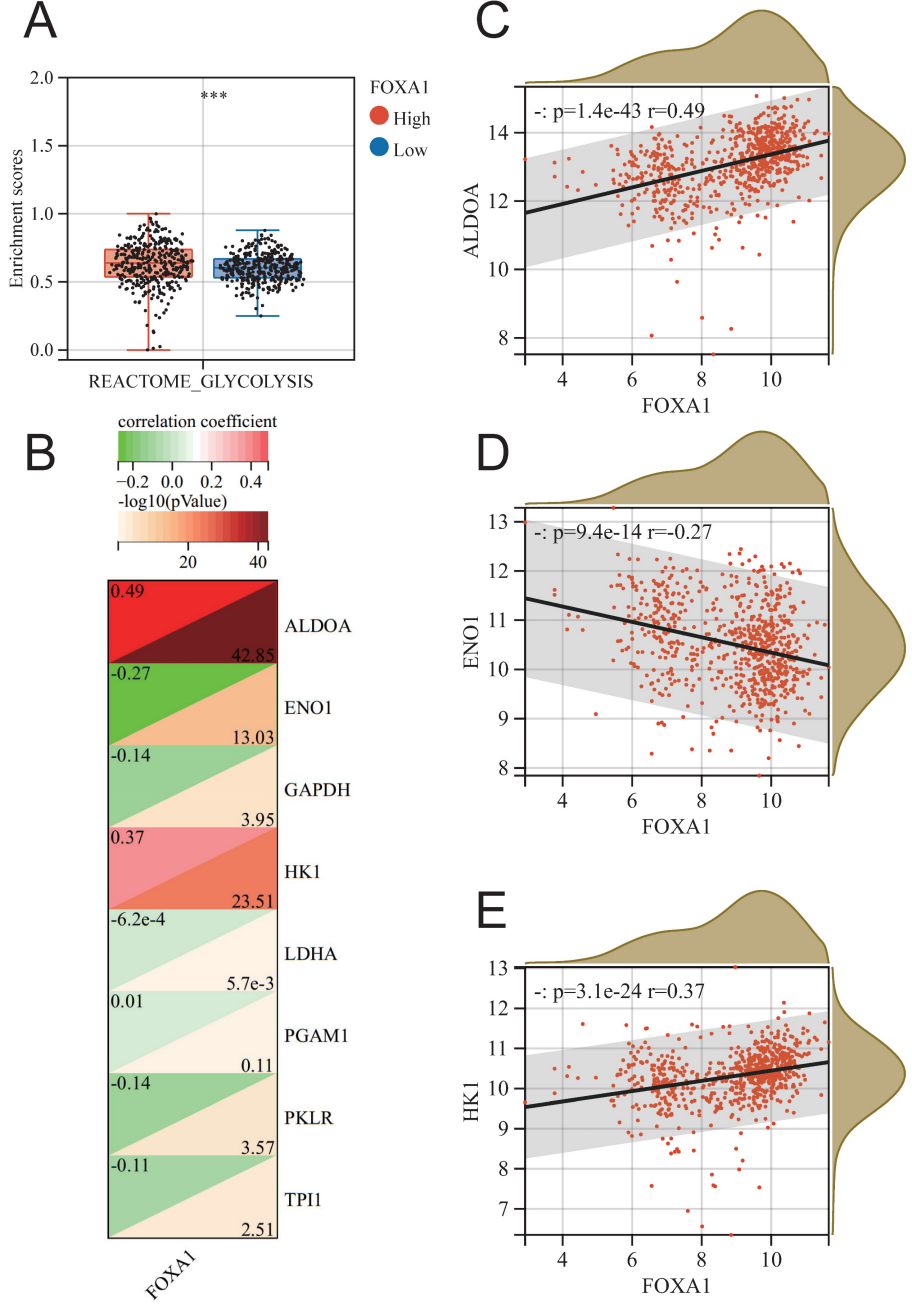
The relationship between FOXA1 and glycolysis. (A) The enrichment level of the glycolysis pathway between the high FOXA1 group and the low FOXA1 group. (B) The correlation between the expression levels of key genes in the glycolysis pathway and FOXA1. FOXA1 is significantly positively correlated with ALDOA (C), negatively correlated with ENO1 (D), and positively correlated with HK1 (E).

In order to further identify the potential biological function of FOXA1 in breast cancer, we conducted GSEA analysis of KEGG pathway based on FOXA1 expression in merge data, and found 19 pathways with significant changes (Supplementary Table 6). Exploring the changes in pathway enrichment after knockdown of FOXA1 gene using the GPSA tool, a total of 44 KEGG pathways were enriched (Supplementary Table 7). Overall, three pathways, including OXIDATIVE PHOSPHORYLATION, AUTOIMMUNE THYROID DISEASE and PEROXISOME, showed significant differences in enrichment in both GSEA analysis using FOXA1 gene expression levels and FOXA1 gene knockdown (Fig. 7B). PEROXISOME pathway was significantly enriched in BC samples with high FOXA1 expression, while OXIDATIVE PHOSPHORYLATION and AUTOIMMUNE THYROID DISEASE pathways were significantly enriched in BC samples with low FOXA1 expression (Fig. 7C). Consistently, the OXIDATIVE PHOSPHORYLATION and AUTOIMMUNE THYROID DISEASE pathways were significantly upregulated after knockdown of FOXA1 in cells, while the PEROXISOME pathway was significantly downregulated after knockdown of FOXA1 in cells (Fig. 7D-7F). In addition, the pan-cancer analysis suggests that the activation of these three pathways is also significantly correlated with FOXA1 in other cancer types. In the TCGA database, the PEROXISOME pathway was also significantly enriched in BC samples with high FOXA1 expression, while the OXIDATIVE PHOSPHORYLATION and AUTOIMMUNE THYROID DISEASE pathways were also significantly enriched in BC samples with low FOXA1 expression; this is consistent with our results obtained from the merge data. Previous literature suggests that the reduction of oxidative phosphorylation is closely related to the peroxisome and glycolysis in tumor cells^38^-^40^. Therefore, we further explored the correlation between FOXA1 gene expression and the activation of glycolysis pathway (Fig. 8A). Based on the median value, the BC patients in the merge data were divided into the FOXA1 high group and the FOXA1 low group. The FOXA1 high group had a higher degree of activation of the glycolysis pathway. Furthermore, FOXA1 was significantly positively correlated with the key genes ALDOA and HK1 of glycolysis, and was significantly negatively correlated with ENO1 (Fig. 8B-8E).

### FOXA1 affects the homologous recombination repair (HRR) status of BC tumor

**Figure 9:**
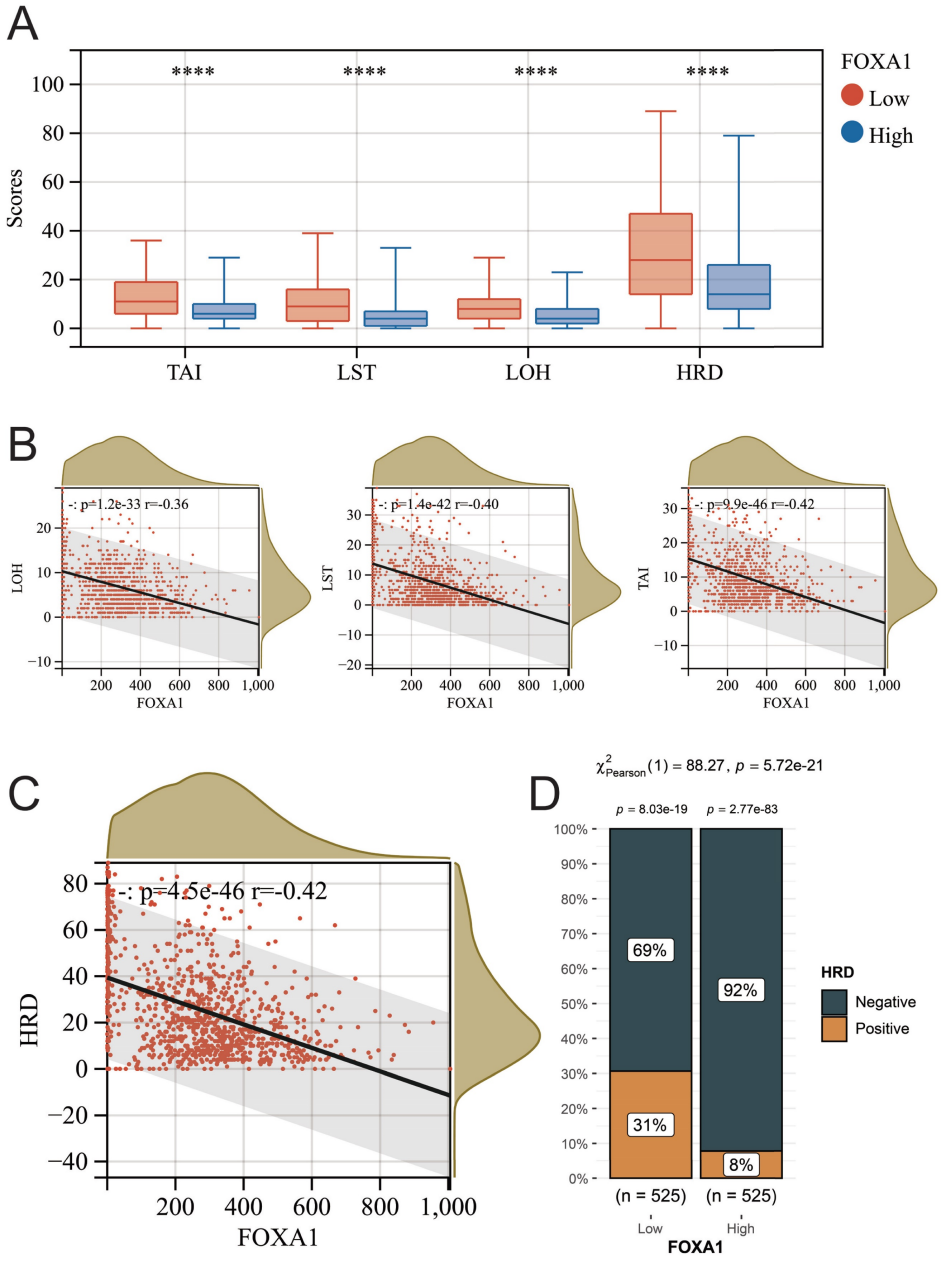
The relationship between FOXA1 and HRD. (A) The TAI, LST, LOH, and HRD scores between the high FOXA1 group and the low FOXA1 group. FOXA1 is significantly negatively correlated with TAI, LST, LOH (B), and HRD (C) scores. (D) The proportion of HRD-positive and HRD-negative breast cancer patients between the high FOXA1 group and the low FOXA1 group.

The relationship between tumor homologous recombination deficiency (HRD) and chemotherapy efficacy has been widely investigated. Therefore, we further explored the relationship between FOXA1 and HRD in BC tumors. In recent times, a novel biomarker, the HRD score, has been introduced, grounded on distinctive genomic scar signatures to detect Homologous Recombination Deficiency (HRD), independent of its etiological or mechanistic origins. The HRD score represents an unweighted summation of three pivotal metrics: Loss of Heterozygosity (LOH), Large-Scale State Transitions (LST), and Telomeric Allelic Imbalances (TAI), mathematically expressed as HRD = LOH + LST + TAI. This approach offers a comprehensive assessment of HRD status. We obtained the HRD scores of all samples in TCGA-BRCA from previous literature (Supplementary Table 8)^41^. The higher the score, the more severe the defect in the HRR pathway. Regardless of whether it pertains to HRD, TAI, LST, or LOH scores, a significant reduction is observed in BC samples that exhibit high expression of FOXA1. Furthermore, there exists a noteworthy negative correlation between these scores and the level of FOXA1 gene expression (Fig. 9A-9C). According to previous literature, BC patients with HRD scores greater than 42 are defined as HRD-positive, and the proportion of HRD-positive patients in BC patients with low expression of FOXA1 is significantly reduced (Fig. 9D). In summary, FOXA1 can protect the function of the HRR pathway and reduce genomic instability in BC patients.

### Pan-cancer analysis of FOXA1

FOXA1 exhibits differential expression in numerous tumor samples, with up-regulated expression levels in tumor tissues of CEST, LUAD, BRCA, STAD, PRAD, and PAAD, and down-regulated expression levels in tumor tissues of COAD, KIRP, HNSC, and KICH (Supplementary Fig. 7A). In renal tumors, such as KIRP and KIRC, FOXA1 expression levels increase with increasing T stage (Supplementary Fig. 7B). Compared to KIPAN tumors in stage M0, KIPAN tumors in stage M1 have significantly higher expression of FOXA1; while in colorectal cancer, COAD and COADREAD, the expression level of FOXA1 in tumors in stage M1 is significantly down-regulated (Supplementary Fig. 7C). In BRCA tumors, the level of FOXA1 is lower in stage N0; whereas in KIRP tumors, the expression level of FOXA1 is significantly upregulated in a sequential manner from stage N0 to N1 to N2 (Supplementary Fig. 7D). It is worth mentioning that in renal tumors, including KIRP, KKIPAN, and KICH, the expression level of FOXA1 in advanced tumor stages III and IV is significantly higher than that in early tumor stages I and II (Supplementary Fig. 7E).

### The experimental validation of the association between FOXA1 and the genomic instability and glycolysis in BC

**Figure 10:**
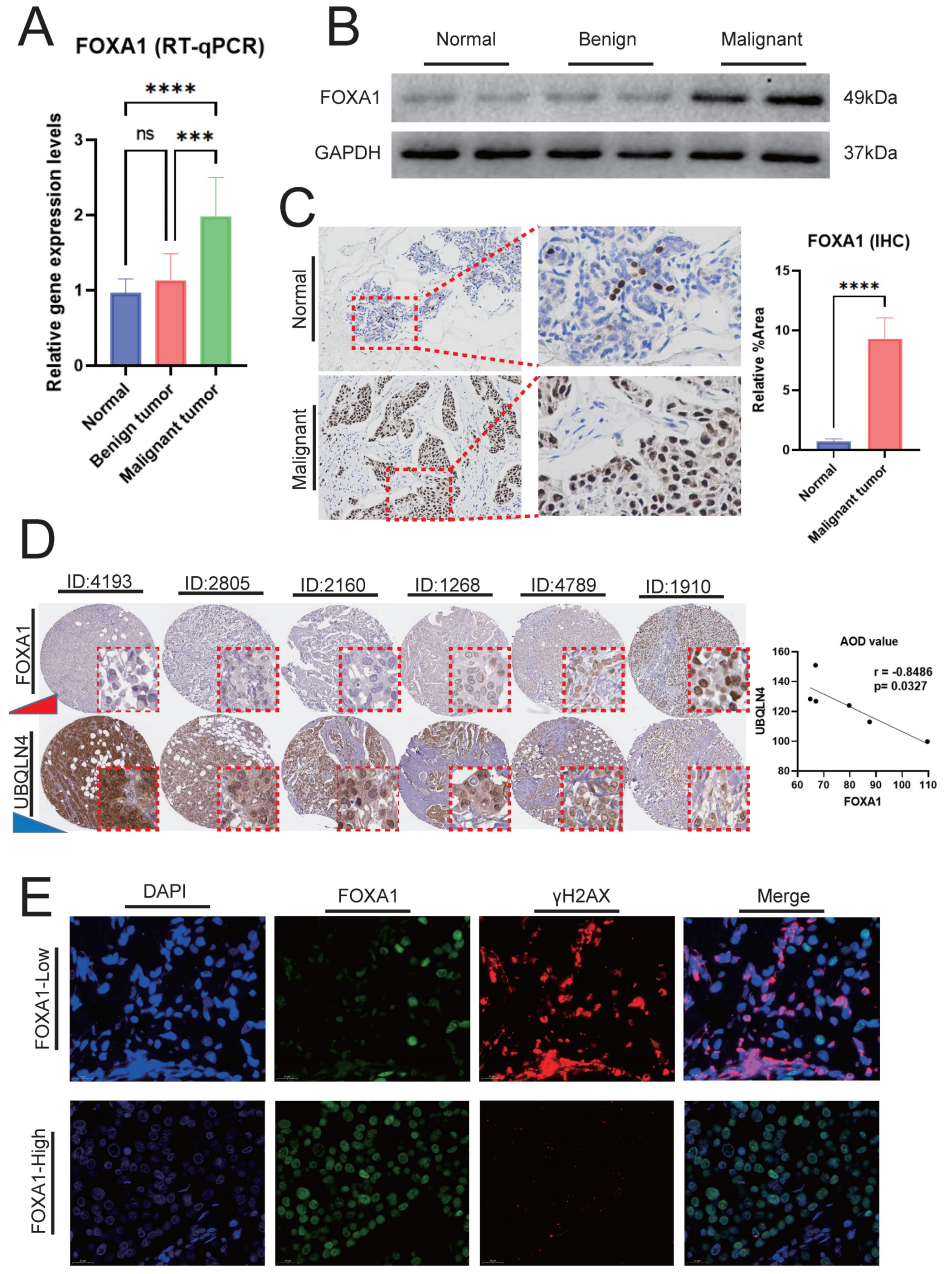
FOXA1 and Its Association with Genomic Instability. RT-qPCR (A) and WB (B) showing the mRNA and protein expression levels of FOXA1 in BC tumors, adjacent normal breast tissues, and benign breast tumors, respectively. (C) IHC analysis revealing the differences in the number of FOXA1-positive cells between BC and adjacent normal breast tissues. (D) Correlation between FOXA1 and UBQLN4 protein expression levels in the same patient in the HPA database. (E) Immunofluorescence showing the correlation between the expression levels of FOXA1 and γH2AX proteins.

In BC, the gene expression levels and protein expression levels of FOXA1 were significantly higher compared to those in adjacent normal breast tissues and benign breast tumors (Fig. 10A-10B). IHC analysis reveals that the number of FOXA1-positive cells in BC is substantially higher than that in adjacent normal tissues (Fig. 10C). Our findings above indicated a significant negative correlation between FOXA1 and HRD characteristics in BC, wherein HRD represents a crucial form of genomic instability. In the TCGA-BRCA cohort, the majority of the top ten genes with the highest mutation rates were significantly more mutated in patients with low FOXA1 expression (Supplementary Fig. 8A). Additionally, four tumor gene mutation-related indicators, including APOBEC Enrichment score, MATH score, TCW score, and TMB score, were all significantly higher in patients with low FOXA1 expression (Supplementary Fig. 8B). This suggested that patients with low FOXA1 expression bear a higher frequency of gene mutations.

Utilizing the HPA database, we conducted a comprehensive search for BC patients with both FOXA1 and UBQLNA (a key marker of genomic instability, with increasing expression levels correlating with higher degrees of genomic instability) immunohistochemical data. Our analysis revealed a decrease in UBQLNA expression with increasing levels of FOXA1 protein expression, demonstrating a significant negative correlation between the AOD values of FOXA1 and UBQLNA (Fig. 10D). To further validate our findings, we employed immunofluorescence to investigate the correlation between FOXA1 and γH2AX, a phosphorylated protein whose robust positivity indicates a higher level of genomic instability. High levels of γH2AX were observed in BC cells with weak or negative FOXA1 expression, whereas a significant reduction in γH2AX levels was noted in BC cells exhibiting strong FOXA1 positivity (Fig. 10E). ECAR and OCR are indicators of glycolysis, reflecting the metabolic status of glycolysis. The Seahorse XF extracellular flux analyzer revealed that the downregulation of FOXA1 in BC cells decreased the level of ECAR while increasing the level of OCR (Supplementary Fig. 9A-9B). Furthermore, WB analysis revealed that the expression levels of glycolysis-related molecular markers were decreased following the knockdown of FOXA1 (Supplementary Fig. 9C). Therefore, FOXA1 enhances the potential of BC cells to undergo glycolysis.

### Predictive Potential of FOXA1 for Sensitivity to PARP Inhibitors

**Figure 11:**
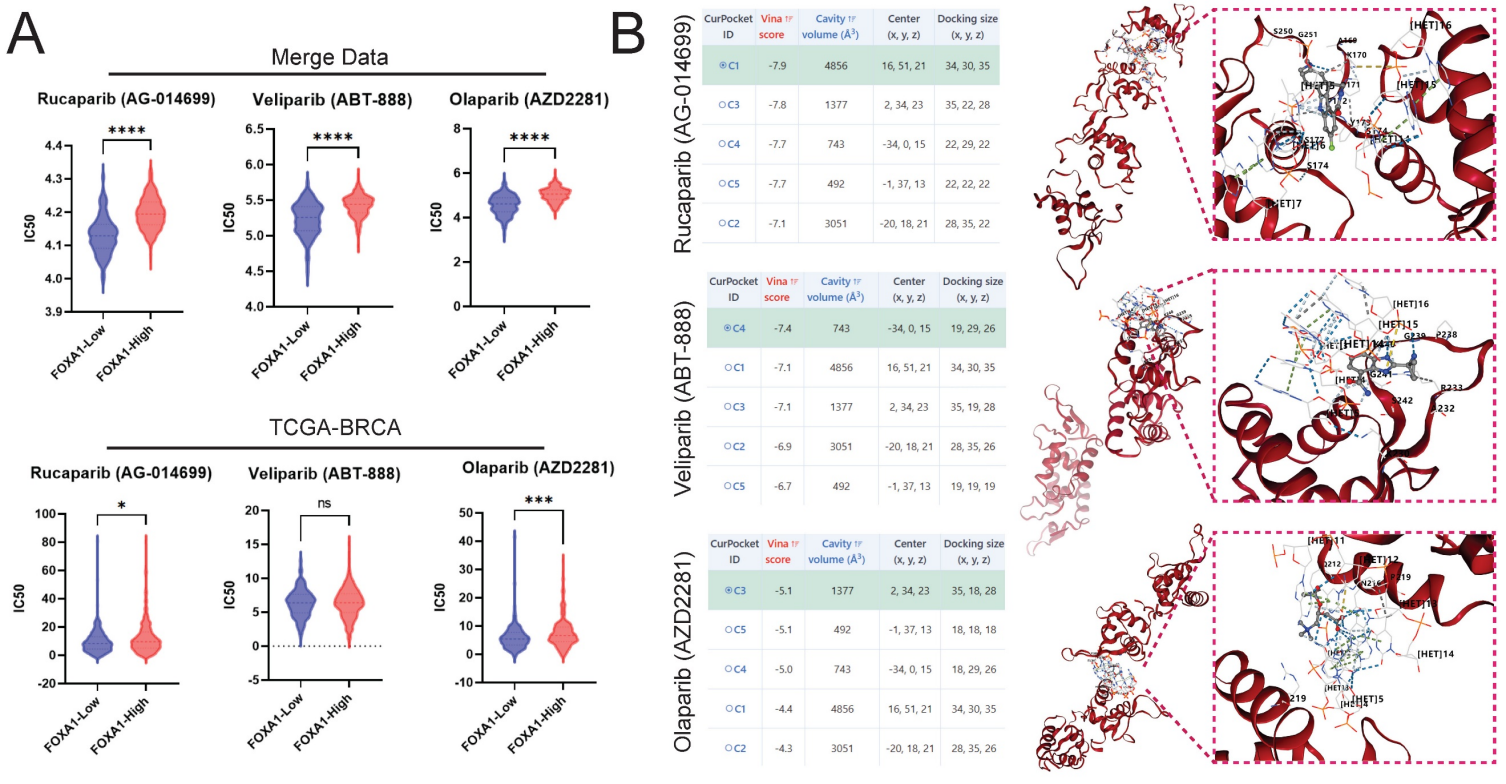
Drug Sensitivity Prediction and Molecular Docking. Prediction of PARP Inhibitor IC50 in High and Low FOXA1 Expression Groups in merge data (A) and TCGA-BRCA Using the pRRophetic Algorithm. (B) Docking of PARP Inhibitors with FOXA1 using CB-Dock2 and displaying the Top Five Docking Scores and the Conformation of the Highest Scoring Docking Pose.

The pRRophetic algorithm suggested that BC patients with low FOXA1 expression are more sensitive to PARP inhibitors, both in the merge data and the TCGA-BRCA cohort (Fig. 11A). Molecular docking suggested that PARP inhibitors and FOXA1 protein have a good interaction; the best docking scores of Rucaparib, Veliparib, and Olaparib with FOXA1 were -7.9, -7.4, and -5.1, respectively (Fig. 11B).

## Discussion

The response of advanced-stage BC patients to NAC is vital for predicting tumor recurrence and prognosis^42^, ^43^. Exploring new gene biomarkers and their biological mechanisms to predict NAC response holds significant clinical importance. However, previous studies primarily examined post-surgical tumor samples, with limited research analyzing pre-chemotherapy samples obtained through FNA. Our study integrated five high-throughput sequencing data obtained through FNA and identified 11 genes as biomarkers for predicting NAC response through FNA. We also validated FOXA1 as a key gene regulating BC glycolysis and genomic instability, affecting NAC response.

Sequencing data from large surgical specimens is typically more stable and reliable. However, for high-throughput sequencing data from FNA samples, special attention may be needed during data analysis to ensure the reliability of the results, due to potential noise and bias in the data. Additionally, the limited DNA or RNA quantity in FNA samples may impose restrictions on sequencing depth, potentially affecting the detection of certain low-frequency variations. Hence, biomarkers derived from sequencing data of large surgical samples in previous studies have limitations when applied to FNA samples. However, FNA is one of the simplest techniques for obtaining BC tissue samples before chemotherapy. Therefore, it is necessary and beneficial to develop new treatment-sensitive biomarkers using FNA data, which can contribute to the development of personalized treatment plans for patients.

Through various bioinformatics techniques, we identified FOXA1 as a biomarker for NAC sensitivity in BC patients using FNA samples. FOXA1, also known as HNF3A or hepatocyte nuclear factor 3-alpha, is a crucial transcription factor located on the human 14q21.1 chromosome^44^, ^45^. It is a member of the FOX family and plays a significant role in gene transcription regulation. FOXA1 has the ability to directly bind to chromatin, opening up tightly packed chromatin structures. This assists in the binding of other transcription factors such as the estrogen receptor (ER) and the androgen receptor (AR), and is essential in gene transcription regulation^46^, ^47^. Our study revealed that knocking down FOXA1 reduces the glycolysis level in breast cancer. Yanfei Zhang's research has demonstrated that the targeted knockdown of TEX19 results in a substantial reduction in the levels of pyruvate, lactate, citrate, and malate, indicating its pivotal role in regulating metabolic processes^48^. Furthermore, the upregulation of TEX19 was found to potentiate glycolysis in lung adenocarcinoma, emphasizing its pro-metabolic function in this context. To elucidate the underlying mechanisms, luciferase reporter assays and chromatin immunoprecipitation (ChIP) experiments were performed, revealing a direct interaction between FOXA1 and TEX19. This finding suggests that FOXA1 may facilitate glycolysis by enhancing the expression of TEX19, thereby providing novel insights into the complex regulatory network governing cancer metabolism. Furthermore, Jiangtao Pu et al. found that FOXA1 can mediate immune escape in lung adenocarcinoma by upregulating UBE2T, which in turn promotes glycolysis^49^. However, the interactions between FOXA1 and glycolysis mentioned above have been confirmed in lung adenocarcinoma. Our study is the first to discover that FOXA1 promotes the metabolic reprogramming of glycolysis in BC cells. Furthermore, a limitation of our study stems from the incomplete collection of patient clinical staging and ER/PR status across most of the cohorts included. Consequently, we were unable to conduct stratified analyses based on these critical clinical parameters, which could have provided deeper insights into the study findings.

Moreover, our study is the first to explore and validate that the loss of FOXA1 increases genomic instability in BC. Previous studies have indicated a high expression of FOXA1 in various tumor tissues and its pivotal role in biological processes such as the cell cycle, epithelial differentiation, and metabolism, suggesting its potential association with genomic instability in cancer cells^50^-^54^. UBQLN4 serves as a proteasomal shuttle factor involved in various biological processes, including DNA damage repair and maintenance of genomic stability^55^-^58^. Specifically, UBQLN4 is phosphorylated by ATM and interacts with ubiquitylated MRE11, thereby mediating early homologous recombination repair (HRR)^55^. Increased levels of UBQLN4 in tumor cells correlate with heightened genomic instability. Upon stimulation by various physicochemical factors, cellular DNA double-strand breaks prompt phosphorylation modifications at serine 139 on H2AX mediated by kinases such as ATM and ATR, forming phosphorylated H2AX, namely γH2AX^59^-^61^. γH2AX plays a crucial role in DNA damage repair^62^. Hence, the significant inverse correlation between FOXA1 and UBQLN4 with γH2AX suggests the potential involvement of FOXA1 in maintaining genomic stability in BC. A cohort study involving 28 cancer patients has revealed that individuals with reduced expression of Smad4 exhibit a higher overall chemotherapy response^63^. Given Smad4's role as a key gene in upregulating DNA repair and modulating genomic stability, it is plausible that the heightened responsiveness to NAC observed in patients with low FOXA1 expression may also be mediated by genomic instability, despite the need for further experimental validation.

Addtionally, our findings suggested that FOXA1 is advantageous for HRR and reduces the incidence of HRD in BC. HRD diminishes the cellular capacity for DNA repair, compelling tumor cells to rely on alternative repair mechanisms such as error-prone recombination and non-homologous end joining. The aberrant activities of these cellular repair pathways render tumor cells more susceptible to damage by specific chemotherapeutic agents, thereby augmenting the therapeutic efficacy of chemotherapy^64^-^66^. This could partially explain why BC patients with low FOXA1 expression have a higher response to NAC and a higher proportion of pCR patients. PARP inhibitors are a class of pharmaceuticals that can impact the self-replication of cancer cells^1^, ^67^-^69^. They achieve this by inhibiting the DNA damage repair of tumor cells and promoting the apoptosis of tumor cells, thereby enhancing the efficacy of radiotherapy, alkylating agents, and platinum-based chemotherapy. In the event of HRD, the use of PARP inhibitors to suppress PARP function can induce tumor cell death through a synthetic lethality effect^70^, ^71^. Assessing the HRD level of cancer patients is more precise in identifying the population that would benefit from PARP inhibitors than merely testing for the BRCA gene. However, HRD testing is complex and costly. Our study suggested that FOXA1 may serve as a novel biomarker for HRD, warranting further exploration for its potential in predicting breast cancer HRD in future clinical applications. This, in turn, could facilitate HRD detection in clinical settings and reduce treatment costs. Furthermore, FOXA1 can not only serves as a predictive marker for HRD, but also harbors the potential to predict sensitivity to PARP inhibitors treatment. Certainly, another limitation of our study lies in the lack of further experimental investigation into whether FOXA1's predictive capability for PARP inhibitor responsiveness is mediated by its correlation with HRD status, which is an established biomarker for predicting the response to PARP inhibitors. Consequently, our understanding remains incomplete in elucidating the underlying mechanisms linking FOXA1, HRD status, and PARP inhibitor sensitivity. A randomized controlled study has revealed that incorporating veliparib and carboplatin alongside paclitaxel, followed by doxorubicin and cyclophosphamide, enhanced the rate of pCR among patients with triple-negative BC^72^. Consequently, the inclusion of PARP inhibitors could be contemplated as a potential component of NAC for BC patients. Therefore, FOXA1, a biomarker with the potential to simultaneously screen for sensitivity to NAC and PARP inhibitors in breast cancer patients, holds significant clinical translational promise for future clinical application, facilitating the development of personalized treatment regimens. There are several PARP inhibitors at this stage, such as olaparib, niraparib, pamidronate, fluzopamide, lucapari, veliparib, and talazoparib. However, only data for Rucaparib (AG-014699), Veliparib (ABT-888), and Olaparib (AZD2281) are included in the GDSC database, so we cannot calculate the IC50 for other PARP inhibitors.

While this study encompassed comprehensive analyses across multiple databases and research methodologies, it nonetheless presents certain limitations. Primarily, this study is based on public cohorts and did not conduct prospective randomized controlled trials to validate the predictive value of FOXA1 for NAC treatment sensitivity. Additionally, while we identified and validated the relationship between FOXA1 and breast cancer genomic stability and HRD, we did not delve into the underlying mechanisms and associated pathways. Hence, it is imperative for future research to further investigate the specific mechanisms through which FOXA1 influences the sensitivity to NAC and PARP inhibitor treatment. Such exploration can significantly contribute to an enhanced understanding of chemoresistance mechanisms in BC and the development of more personalized treatment regimens.

## Conclusion

In summary, we conducted an analysis of multiple sequencing datasets obtained from various FNA samples using bioinformatics techniques such as WGCNA, machine learning, and GPSA. Then, we identified 11 biomarkers associated with NAC treatment response in BC patients. Furthermore, we discovered that FOXA1 influenced genomic stability, HRD, and glycolysis in BC tumors. Additionally, FOXA1 were found to serve as a biomarker for NAC and PARP inhibitor treatment sensitivity in BC patients, offering new insights and resources for the development of personalized clinical treatment strategies in the future.

## Supplementary Material

Supplementary figures and table legends.

Supplementary tables.
