# Supplementary material for: In Silico Analysis Uncovers FOXA1 as a Potential Biomarker for Predicting Neoadjuvant Chemotherapy Response in Fine-Needle Aspiration Biopsies
Source: J Cancer. 2024 Sep 30;15(18):6052–72. doi: 10.7150/jca.101901 (PMC11493000; doi:10.7150/jca.101901)
Supplement: Supplementary file 1 — Supplementary figures and table legends. [file jcav15p6052s1.pdf]

Supplementary Figure 1: The flowchart of this study.

Supplementary Figure 2: The diagnostic capability of NAC-pCR related genes for breast cancer tumors. (A) Dimensionality-reduction using PCA, t-SNE and UMAP for NAC-pCR related genes. (B) ROC curve of each NAC-pCR related genes diagnosis of BC.

Supplementary Figure 3: Exploration of the biological functions of NAC-pCR related genes. Enrichment scores of 22 tumor-related pathways between RD and pCR patients (A), as well as between cluster C1 and cluster C2 (B). (C) The correlation between the expression levels of NAC-pCR related genes and the enrichment scores of 22 tumor-related pathways.

Supplementary Figure 4: Identification of key genes for NAC treatment response. SVM (A), xgboost (B), LASSO (C) for key gene selection. In the D2558 dataset of the GPSA database, the enrichment scores of the gene set with increased expression after FOXA1 knockdown are significantly decreased in pCR patients (D), while the enrichment scores of the gene set with decreased expression after FOXA1 knockdown are significantly increased in pCR patients (E). (F) The changes in enrichment scores of Hallmarkers pathways in pCR patients show a significant negative correlation with the changes in enrichment scores of Hallmarkers pathways after FOXA1 knockdown.

Supplementary Figure 5: Identification of genes that may be involved in the response of breast cancer patients to NAC treatment based on GPSA analysis. (A) The top 20 genes with the highest GPSI scores in the GPSA analysis. (B) The changes in

enrichment scores of the Hallmarker pathways after genetic perturbation of the top 20 genes with the highest GPSI scores. (C) The percentage of times each hallmark gene set was enriched.

Supplementary Figure 6: Exploration of the expression of FOXA1 in breast cancer. (A) The expression levels of FOXA1 in breast cancer tumors and normal tissues In the GEPIA database. (B) The protein expression levels of FOXA1 in breast cancer tumors and normal tissues in the HPA database. (C) The protein localization of FOXA1 in tumor cells in the HPA database. (D) Spatial transcriptomics data demonstrate the localization of FOXA1-positive cells and the immune cell infiltration around these cells.

Supplementary Figure 7: A pan-cancer analysis of FOXA1. (A) The expression levels of FOXA1 between different cancers and their adjacent normal tissues in the TCGA database. The expression levels of FOXA1 among different cancers with different T stages (B), M stages (C), and N stages (D).

Supplementary Figure 8: FOXA1 and the Frequency of Gene Mutations in Breast Cancer. (A) The Difference in Mutation Frequencies of the Top Ten Genes with the Highest Overall Mutation Rates in Breast Cancer between FOXA1 High/Low Expression Groups. (B) Levels of Four Tumor Gene Mutation-Related Indicators between FOXA1 High/Low Expression Groups

Supplementary Figure 9: The relationship between FOXA1 and glycolysis. The Seahorse XF Extracellular Flux Analyzers were utilized to assess the levels of ECAR (A) and OCR (B) within cellular environments. (C) WB showing the expression levels of glycolysis-related proteins (HK2, GLUT1, and LDHA).

Supplementary Table 1: The clinical information of patients in GEO datasets.

Supplementary Table 2: The genes in the 14 co-expression gene modules.

Supplementary Table 3: Hub genes in black module and brown module.

Supplementary Table 4: Genes in 22 tumor-related pathways.

Supplementary Table 5: The GPSA algorithm identified a total of 275 gene perturbations related to achieving pCR with NAC treatment.

Supplementary Table 6: GSEA analysis of KEGG pathway based on FOXA1 expression in merge data.

Supplementary Table 7: Exploring the changes in pathway enrichment after knockdown of FOXA1 gene using the GPSA tool.

Supplementary Table 8: HRD scores of all samples in TCGA-BRCA.

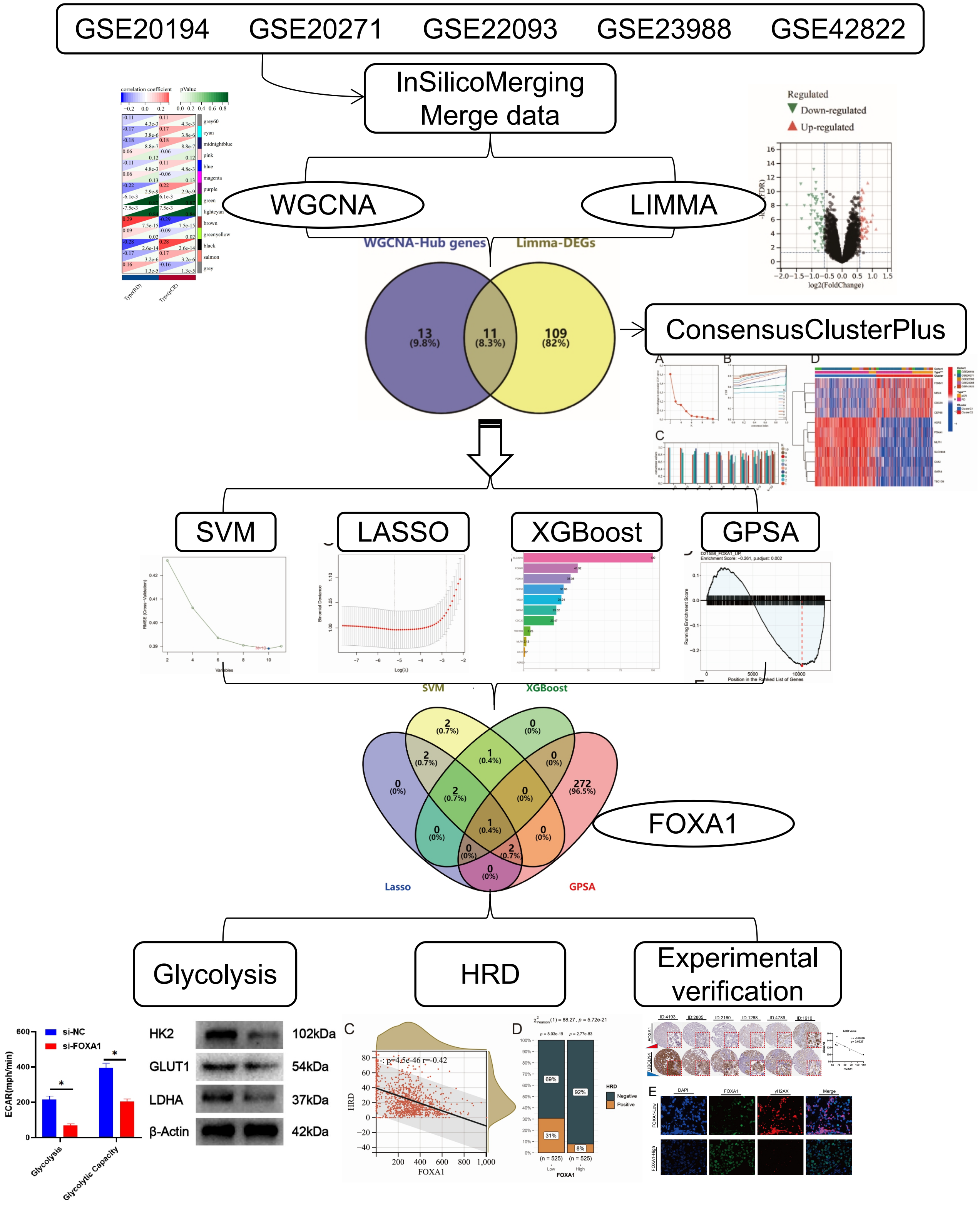

GSE20194
GSE20271
GSE22093
GSE23988
GSE42822
InSilicoMerging
Merge data
WGCNA
LIMMA
Regulated
Down-regulated
Up-regulated
log2(FoldChange)
WGCNA-Hub genes
Limma-DEGs
13 (9.8%)
11 (8.3%)
109 (82%)
ConsensusClusterPlus
SVM
LASSO
XGBoost
GPSA
SVM
XGBoost
Lasso
GPSA
272 (96.5%)
FOXA1
Glycolysis
HRD
Experimental verification
si-NC
si-FOXA1
ECAR(mph/min)
Glycolysis
Glycolytic Capacity
HK2
GLUT1
LDHA
β-Actin
102kDa
54kDa
37kDa
42kDa
HRD
FOXA1
DAPI
FOXA1
γH2AX
Merge

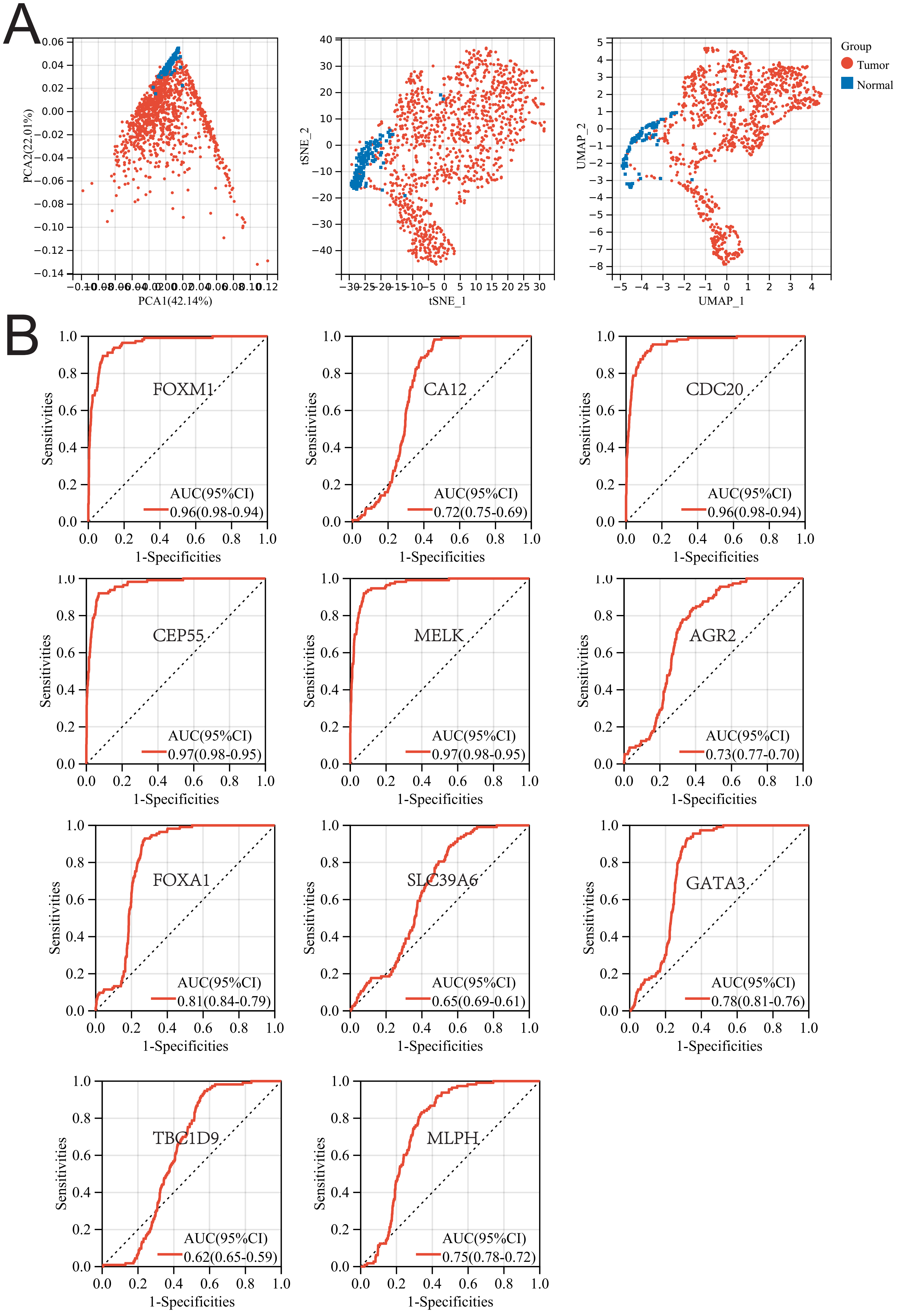

A
Group
Tumor
Normal
PCA1(42.14%)
PCA2(22.01%)
tSNE_1
tSNE_2
UMAP_1
UMAP_2
B
FOXM1
AUC(95%CI)
0.96(0.98-0.94)
CA12
AUC(95%CI)
0.72(0.75-0.69)
CDC20
AUC(95%CI)
0.96(0.98-0.94)
CEP55
AUC(95%CI)
0.97(0.98-0.95)
MELK
AUC(95%CI)
0.97(0.98-0.95)
AGR2
AUC(95%CI)
0.73(0.77-0.70)
FOXA1
AUC(95%CI)
0.81(0.84-0.79)
SLC39A6
AUC(95%CI)
0.65(0.69-0.61)
GATA3
AUC(95%CI)
0.78(0.81-0.76)
TBC1D9
AUC(95%CI)
0.62(0.65-0.59)
MLPH
AUC(95%CI)
0.75(0.78-0.72)
Sensitivities
1-Specificities

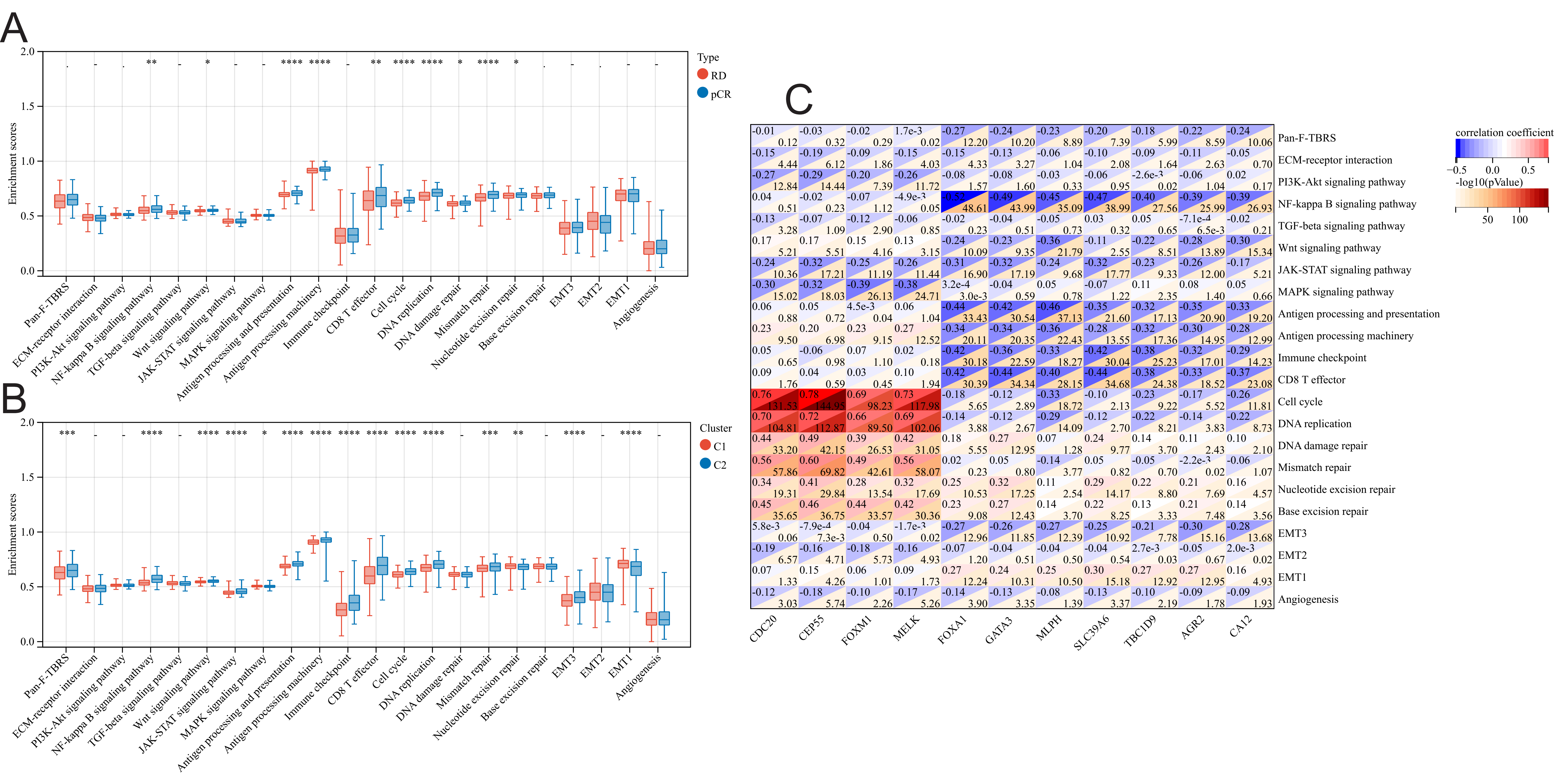
A
B
C
Enrichment scores
Type
RD
pCR
Cluster
C1
C2
Pan-F-TBRS
ECM-receptor interaction
PI3K-Akt signaling pathway
NF-kappa B signaling pathway
TGF-beta signaling pathway
Wnt signaling pathway
JAK-STAT signaling pathway
MAPK signaling pathway
Antigen processing and presentation
Antigen processing machinery
Immune checkpoint
CD8 T effector
Cell cycle
DNA replication
DNA damage repair
Mismatch repair
Nucleotide excision repair
Base excision repair
EMT3
EMT2
EMT1
Angiogenesis
CDC20
CEP55
FOXM1
MELK
FOXA1
GATA3
MLPH
SLC39A6
TBC1D9
AGR2
CA12
correlation coefficient
-log10(pValue)

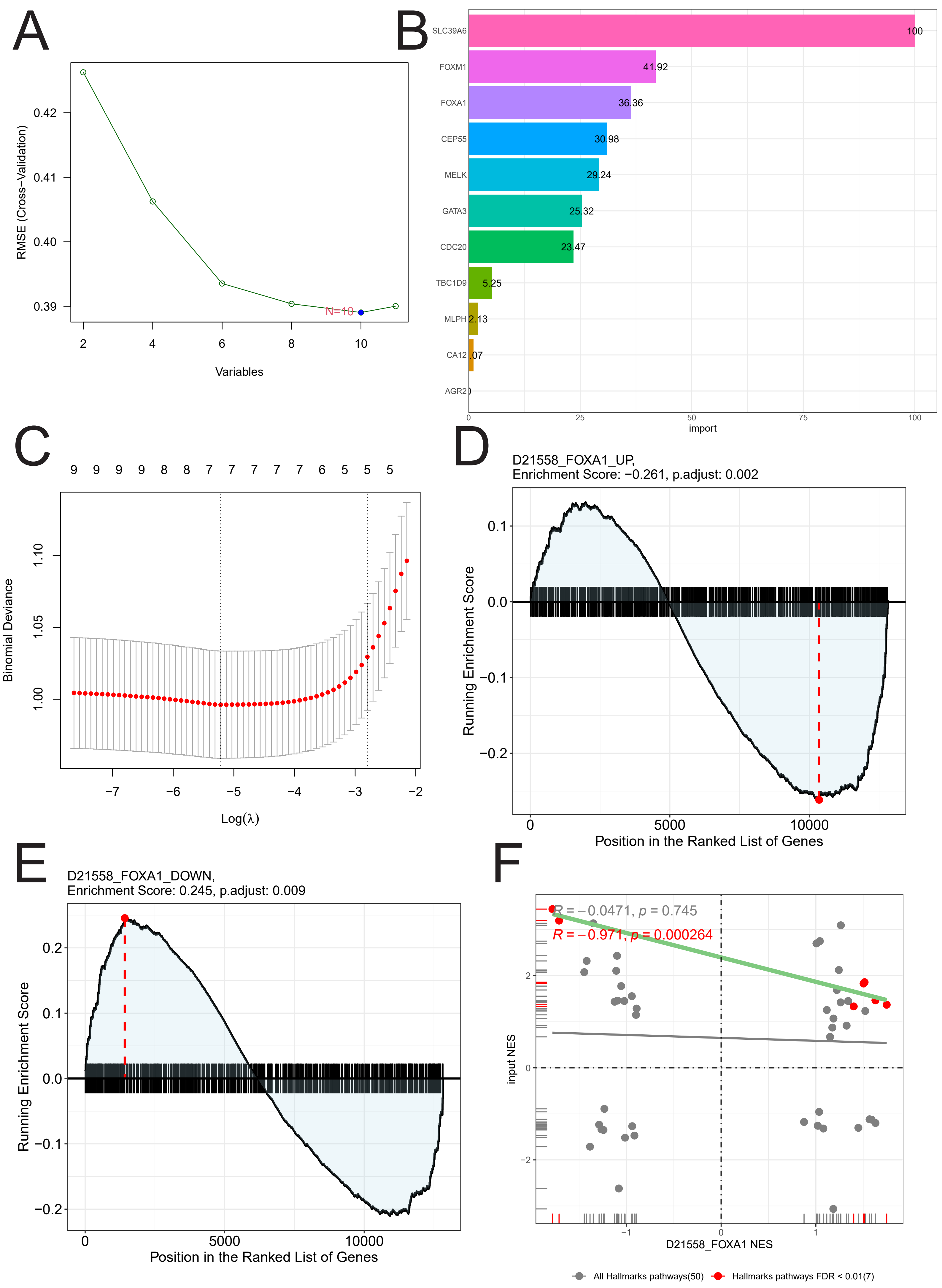
A
RMSE (Cross-Validation)
0.42
0.41
0.40
0.39
N=10
2
4
6
8
10
Variables
B
SLC39A6
FOXM1
FOXA1
CEP55
MELK
GATA3
CDC20
TBC1D9
MLPH
CA12
AGR2
100
41.92
36.36
30.98
29.24
25.32
23.47
5.25
2.13
.07
0
25
50
75
100
import
C
9 9 9 9 8 8 7 7 7 7 7 6 5 5 5
Binomial Deviance
1.10
1.05
1.00
−7 −6 −5 −4 −3 −2
Log(λ)
D
D21558_FOXA1_UP,
Enrichment Score: −0.261, p.adjust: 0.002
Running Enrichment Score
0.1
0.0
−0.1
−0.2
0
5000
10000
Position in the Ranked List of Genes
E
D21558_FOXA1_DOWN,
Enrichment Score: 0.245, p.adjust: 0.009
Running Enrichment Score
0.2
0.1
0.0
−0.1
−0.2
0
5000
10000
Position in the Ranked List of Genes
F
R = −0.0471, p = 0.745
R = −0.971, p = 0.000264
input NES
2
0
−2
−1
0
1
D21558_FOXA1 NES
All Hallmarks pathways(50)
Hallmarks pathways FDR < 0.01(7)

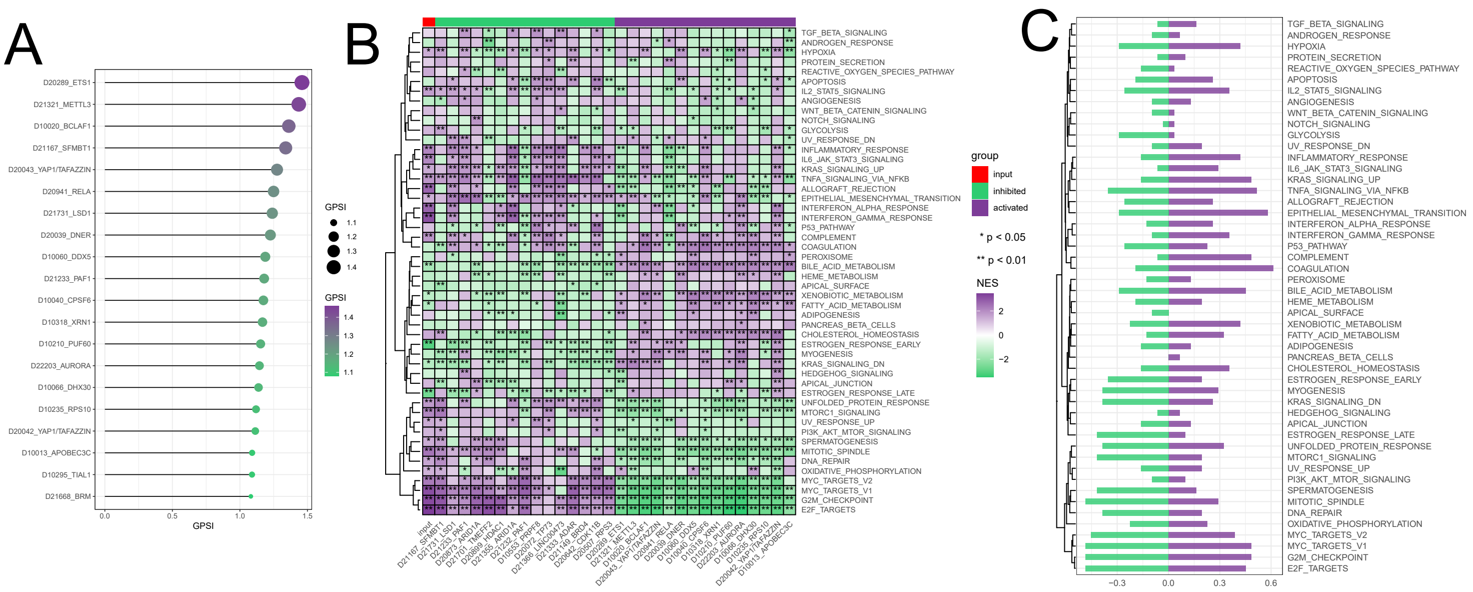
A
D20289_ETS1
D21321_METTL3
D10020_BCLAF1
D21167_SFMBT1
D20043_YAP1/TAFAZZIN
D20941_RELA
D21731_LSD1
D20039_DNER
D10060_DDX5
D21233_PAF1
D10040_CPSF6
D10318_XRN1
D10210_PUF60
D22203_AURORA
D10066_DHX30
D10235_RPS10
D20042_YAP1/TAFAZZIN
D10013_APOBEC3C
D10295_TIAL1
D21668_BRM
0.0
0.5
1.0
1.5
GPSI
GPSI
1.1
1.2
1.3
1.4
GPSI
1.4
1.3
1.2
1.1
B
TGF_BETA_SIGNALING
ANDROGEN_RESPONSE
HYPOXIA
PROTEIN_SECRETION
REACTIVE_OXYGEN_SPECIES_PATHWAY
APOPTOSIS
IL2_STAT5_SIGNALING
ANGIOGENESIS
WNT_BETA_CATENIN_SIGNALING
NOTCH_SIGNALING
GLYCOLYSIS
UV_RESPONSE_DN
INFLAMMATORY_RESPONSE
IL6_JAK_STAT3_SIGNALING
KRAS_SIGNALING_UP
TNFA_SIGNALING_VIA_NFKB
ALLOGRAFT_REJECTION
EPITHELIAL_MESENCHYMAL_TRANSITION
INTERFERON_ALPHA_RESPONSE
INTERFERON_GAMMA_RESPONSE
P53_PATHWAY
COMPLEMENT
COAGULATION
PEROXISOME
BILE_ACID_METABOLISM
HEME_METABOLISM
APICAL_SURFACE
XENOBIOTIC_METABOLISM
FATTY_ACID_METABOLISM
ADIPOGENESIS
PANCREAS_BETA_CELLS
CHOLESTEROL_HOMEOSTASIS
ESTROGEN_RESPONSE_EARLY
MYOGENESIS
KRAS_SIGNALING_DN
HEDGEHOG_SIGNALING
APICAL_JUNCTION
ESTROGEN_RESPONSE_LATE
UNFOLDED_PROTEIN_RESPONSE
MTORC1_SIGNALING
UV_RESPONSE_UP
PI3K_AKT_MTOR_SIGNALING
SPERMATOGENESIS
MITOTIC_SPINDLE
DNA_REPAIR
OXIDATIVE_PHOSPHORYLATION
MYC_TARGETS_V2
MYC_TARGETS_V1
G2M_CHECKPOINT
E2F_TARGETS
input
D21167_SFMBT1
D21731_LSD1
D21233_PAF1
D20873_ARID1A
D21701_TMEFF2
D20899_HDAC1
D21355_ARID1A
D21232_PAF1
D10553_PRPF8
D20072_TP73
D21366_LINC00473
D21333_ADAR
D21149_BRD4
D20642_CDK11B
D20507_RPS3
D20289_ETS1
D21321_METTL3
D10020_BCLAF1
D20043_YAP1/TAFAZZIN
D20941_RELA
D20039_DNER
D10060_DDX5
D10040_CPSF6
D10318_XRN1
D10210_PUF60
D22203_AURORA
D10066_DHX30
D10235_RPS10
D20042_YAP1/TAFAZZIN
D10013_APOBEC3C
group
input
inhibited
activated
* p < 0.05
** p < 0.01
NES
2
0
−2
C
TGF_BETA_SIGNALING
ANDROGEN_RESPONSE
HYPOXIA
PROTEIN_SECRETION
REACTIVE_OXYGEN_SPECIES_PATHWAY
APOPTOSIS
IL2_STAT5_SIGNALING
ANGIOGENESIS
WNT_BETA_CATENIN_SIGNALING
NOTCH_SIGNALING
GLYCOLYSIS
UV_RESPONSE_DN
INFLAMMATORY_RESPONSE
IL6_JAK_STAT3_SIGNALING
KRAS_SIGNALING_UP
TNFA_SIGNALING_VIA_NFKB
ALLOGRAFT_REJECTION
EPITHELIAL_MESENCHYMAL_TRANSITION
INTERFERON_ALPHA_RESPONSE
INTERFERON_GAMMA_RESPONSE
P53_PATHWAY
COMPLEMENT
COAGULATION
PEROXISOME
BILE_ACID_METABOLISM
HEME_METABOLISM
APICAL_SURFACE
XENOBIOTIC_METABOLISM
FATTY_ACID_METABOLISM
ADIPOGENESIS
PANCREAS_BETA_CELLS
CHOLESTEROL_HOMEOSTASIS
ESTROGEN_RESPONSE_EARLY
MYOGENESIS
KRAS_SIGNALING_DN
HEDGEHOG_SIGNALING
APICAL_JUNCTION
ESTROGEN_RESPONSE_LATE
UNFOLDED_PROTEIN_RESPONSE
MTORC1_SIGNALING
UV_RESPONSE_UP
PI3K_AKT_MTOR_SIGNALING
SPERMATOGENESIS
MITOTIC_SPINDLE
DNA_REPAIR
OXIDATIVE_PHOSPHORYLATION
MYC_TARGETS_V2
MYC_TARGETS_V1
G2M_CHECKPOINT
E2F_TARGETS
−0.3
0.0
0.3
0.6

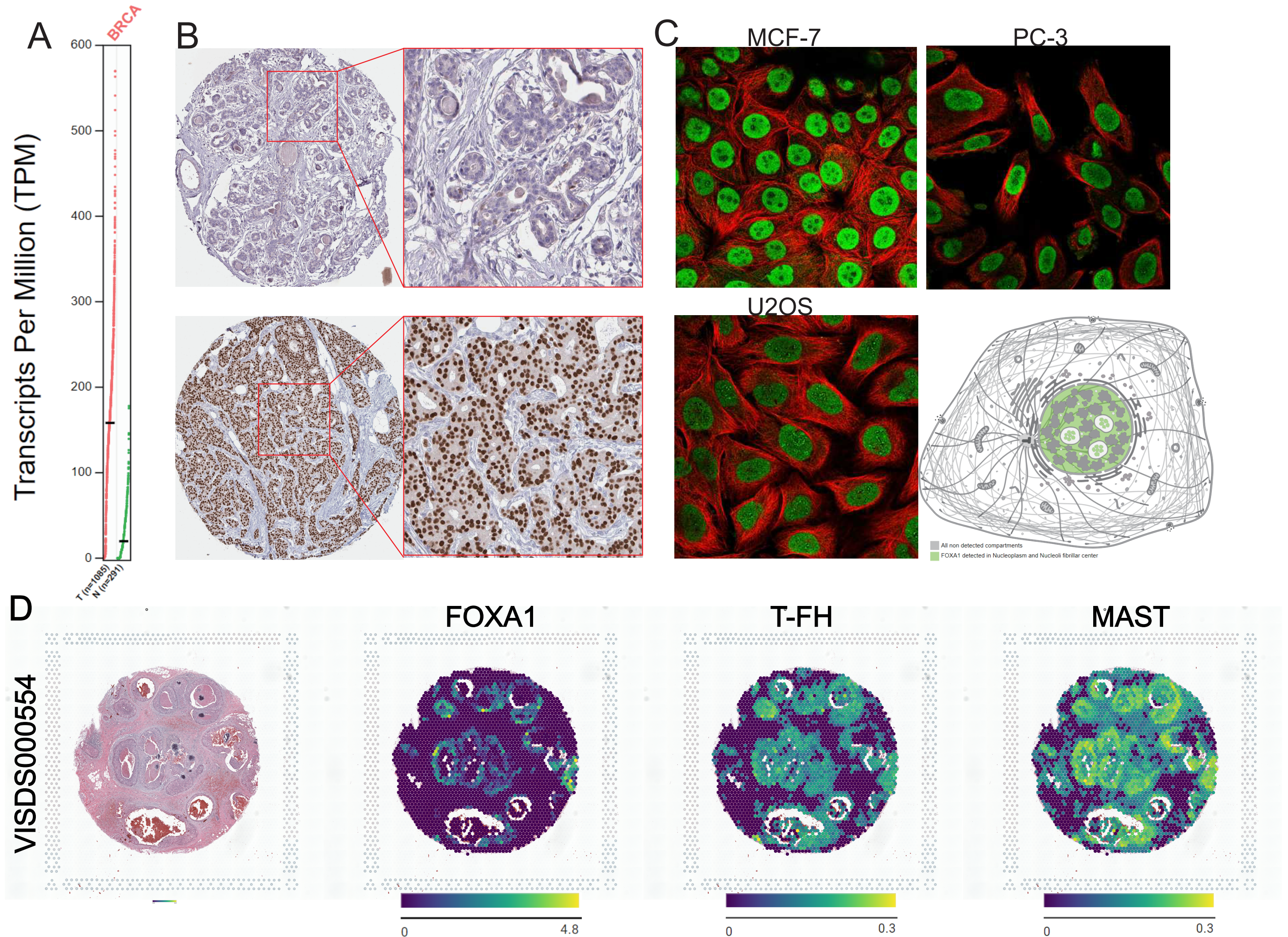
A
BRCA
Transcripts Per Million (TPM)
600
500
400
300
200
100
0
T (n=1085)
N (n=291)
B
C
MCF-7
PC-3
U2OS
All non detected compartments
FOXA1 detected in Nucleoplasm and Nucleoli fibrillar center
D
VISDS000554
FOXA1
T-FH
MAST
0
4.8
0
0.3
0
0.3

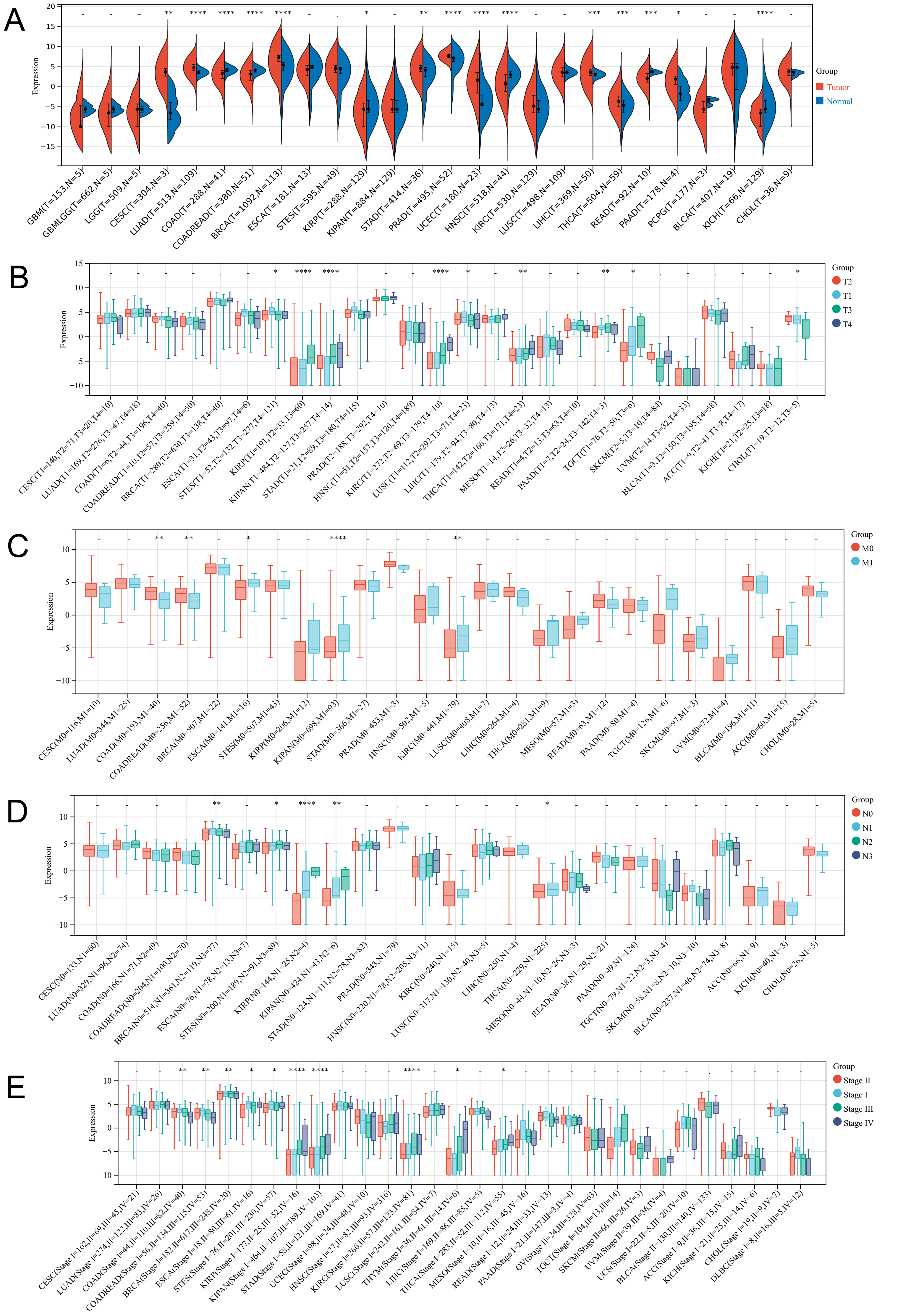

A
Expression
Group
Tumor
Normal
GBM(T=153,N=5)
GBMLGG(T=662,N=5)
LGG(T=509,N=5)
CESC(T=304,N=3)
LUAD(T=513,N=109)
COAD(T=288,N=41)
COADREAD(T=380,N=51)
BRCA(T=1092,N=113)
ESCA(T=181,N=13)
STES(T=595,N=49)
KIRP(T=288,N=129)
KIPAN(T=884,N=129)
STAD(T=414,N=36)
PRAD(T=495,N=52)
UCEC(T=180,N=23)
HNSC(T=518,N=44)
KIRC(T=530,N=129)
LUSC(T=498,N=109)
LIHC(T=369,N=50)
THCA(T=504,N=59)
READ(T=92,N=10)
PAAD(T=178,N=4)
PCPG(T=177,N=3)
BLCA(T=407,N=19)
KICH(T=66,N=129)
CHOL(T=36,N=9)
B
Expression
Group
T2
T1
T3
T4
CESC(T1=140,T2=71,T3=20,T4=10)
LUAD(T1=169,T2=276,T3=47,T4=18)
COAD(T1=6,T2=44,T3=196,T4=40)
COADREAD(T1=10,T2=57,T3=259,T4=50)
BRCA(T1=280,T2=630,T3=138,T4=40)
ESCA(T1=31,T2=43,T3=97,T4=6)
STES(T1=52,T2=132,T3=277,T4=121)
KIRP(T1=191,T2=33,T3=60)
KIPAN(T1=484,T2=127,T3=257,T4=14)
STAD(T1=21,T2=89,T3=180,T4=115)
PRAD(T2=188,T3=292,T4=10)
HNSC(T1=51,T2=157,T3=120,T4=189)
KIRC(T1=272,T2=69,T3=179,T4=10)
LUSC(T1=112,T2=292,T3=71,T4=23)
LIHC(T1=179,T2=94,T3=80,T4=13)
THCA(T1=142,T2=166,T3=171,T4=23)
MESO(T1=14,T2=26,T3=32,T4=13)
READ(T1=4,T2=13,T3=63,T4=10)
PAAD(T1=7,T2=24,T3=142,T4=3)
TGCT(T1=76,T2=50,T3=6)
SKCM(T2=5,T3=10,T4=84)
UVM(T2=14,T3=32,T4=33)
BLCA(T1=3,T2=150,T3=195,T4=58)
ACC(T1=9,T2=41,T3=8,T4=17)
KICH(T1=21,T2=25,T3=18)
CHOL(T1=19,T2=12,T3=5)
C
Expression
Group
M0
M1
CESC(M0=116,M1=10)
LUAD(M0=344,M1=25)
COAD(M0=193,M1=40)
COADREAD(M0=256,M1=52)
BRCA(M0=907,M1=22)
ESCA(M0=141,M1=16)
STES(M0=507,M1=43)
KIRP(M0=206,M1=12)
KIPAN(M0=698,M1=93)
STAD(M0=366,M1=27)
PRAD(M0=453,M1=3)
HNSC(M0=502,M1=5)
KIRC(M0=441,M1=79)
LUSC(M0=408,M1=7)
LIHC(M0=264,M1=4)
THCA(M0=281,M1=9)
MESO(M0=57,M1=3)
READ(M0=63,M1=12)
PAAD(M0=80,M1=4)
TGCT(M0=126,M1=6)
SKCM(M0=97,M1=3)
UVM(M0=72,M1=4)
BLCA(M0=196,M1=11)
ACC(M0=60,M1=15)
CHOL(M0=28,M1=5)
D
Expression
Group
N0
N1
N2
N3
CESC(N0=133,N1=60)
LUAD(N0=329,N1=96,N2=74)
COAD(N0=166,N1=71,N2=49)
COADREAD(N0=204,N1=100,N2=70)
BRCA(N0=514,N1=361,N2=119,N3=77)
ESCA(N0=76,N1=78,N2=13,N3=7)
STES(N0=200,N1=189,N2=91,N3=89)
KIRP(N0=144,N1=25,N2=4)
KIPAN(N0=424,N1=43,N2=6)
STAD(N0=124,N1=111,N2=78,N3=82)
PRAD(N0=343,N1=79)
HNSC(N0=220,N1=78,N2=205,N3=11)
KIRC(N0=240,N1=15)
LUSC(N0=317,N1=130,N2=40,N3=5)
LIHC(N0=250,N1=4)
THCA(N0=229,N1=225)
MESO(N0=44,N1=10,N2=26,N3=3)
READ(N0=38,N1=29,N2=21)
PAAD(N0=49,N1=124)
TGCT(N0=79,N1=22,N2=3,N3=4)
SKCM(N0=58,N1=8,N2=10,N3=10)
BLCA(N0=237,N1=46,N2=74,N3=8)
ACC(N0=66,N1=9)
KICH(N0=40,N1=3)
CHOL(N0=26,N1=5)
E
Expression
Group
Stage II
Stage I
Stage III
Stage IV
CESC(Stage I=162,II=69,III=45,IV=21)
LUAD(Stage I=274,II=122,III=83,IV=26)
COAD(Stage I=44,II=110,III=82,IV=40)
COADREAD(Stage I=56,II=134,III=115,IV=53)
BRCA(Stage I=182,II=617,III=248,IV=20)
ESCA(Stage I=18,II=80,III=61,IV=16)
STES(Stage I=76,II=201,III=230,IV=57)
KIRP(Stage I=177,II=25,III=52,IV=16)
KIPAN(Stage I=464,II=107,III=189,IV=103)
STAD(Stage I=58,II=121,III=169,IV=41)
UCEC(Stage I=98,II=24,III=48,IV=10)
HNSC(Stage I=27,II=82,III=93,IV=316)
KIRC(Stage I=266,II=57,III=123,IV=81)
LUSC(Stage I=242,II=161,III=84,IV=7)
THYM(Stage I=36,II=61,III=14,IV=6)
LIHC(Stage I=169,II=86,III=85,IV=5)
THCA(Stage I=283,II=52,III=112,IV=55)
MESO(Stage I=10,II=16,III=45,IV=16)
READ(Stage I=12,II=24,III=33,IV=13)
PAAD(Stage I=21,II=147,III=3,IV=4)
OV(Stage II=24,III=328,IV=63)
TGCT(Stage I=104,II=13,III=14)
SKCM(Stage II=66,III=26,IV=3)
UVM(Stage II=39,III=36,IV=4)
UCS(Stage I=22,II=5,III=20,IV=10)
BLCA(Stage II=130,III=140,IV=133)
ACC(Stage I=9,II=36,III=15,IV=15)
KICH(Stage I=21,II=25,III=14,IV=6)
CHOL(Stage I=19,II=9,IV=7)
DLBC(Stage I=8,II=16,III=5,IV=12)

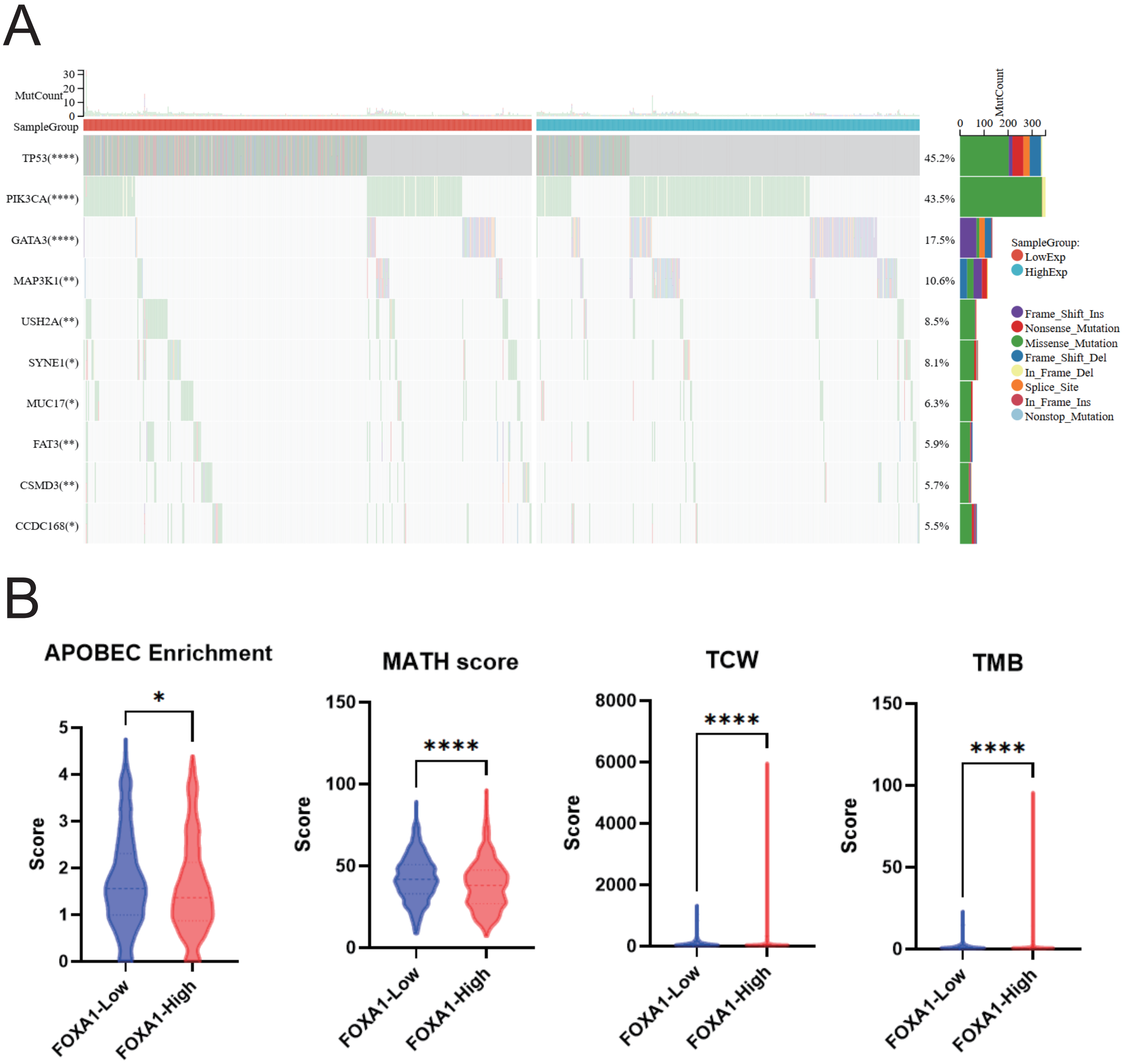
A
MutCount
30
20
10
0
SampleGroup
TP53(****)
PIK3CA(****)
GATA3(****)
MAP3K1(**)
USH2A(**)
SYNE1(*)
MUC17(*)
FAT3(**)
CSMD3(**)
CCDC168(*)
45.2%
43.5%
17.5%
10.6%
8.5%
8.1%
6.3%
5.9%
5.7%
5.5%
MutCount
0 100 200 300
SampleGroup:
LowExp
HighExp
Frame_Shift_Ins
Nonsense_Mutation
Missense_Mutation
Frame_Shift_Del
In_Frame_Del
Splice_Site
In_Frame_Ins
Nonstop_Mutation
B
APOBEC Enrichment
MATH score
TCW
TMB
Score
FOXA1-Low
FOXA1-High
*
****

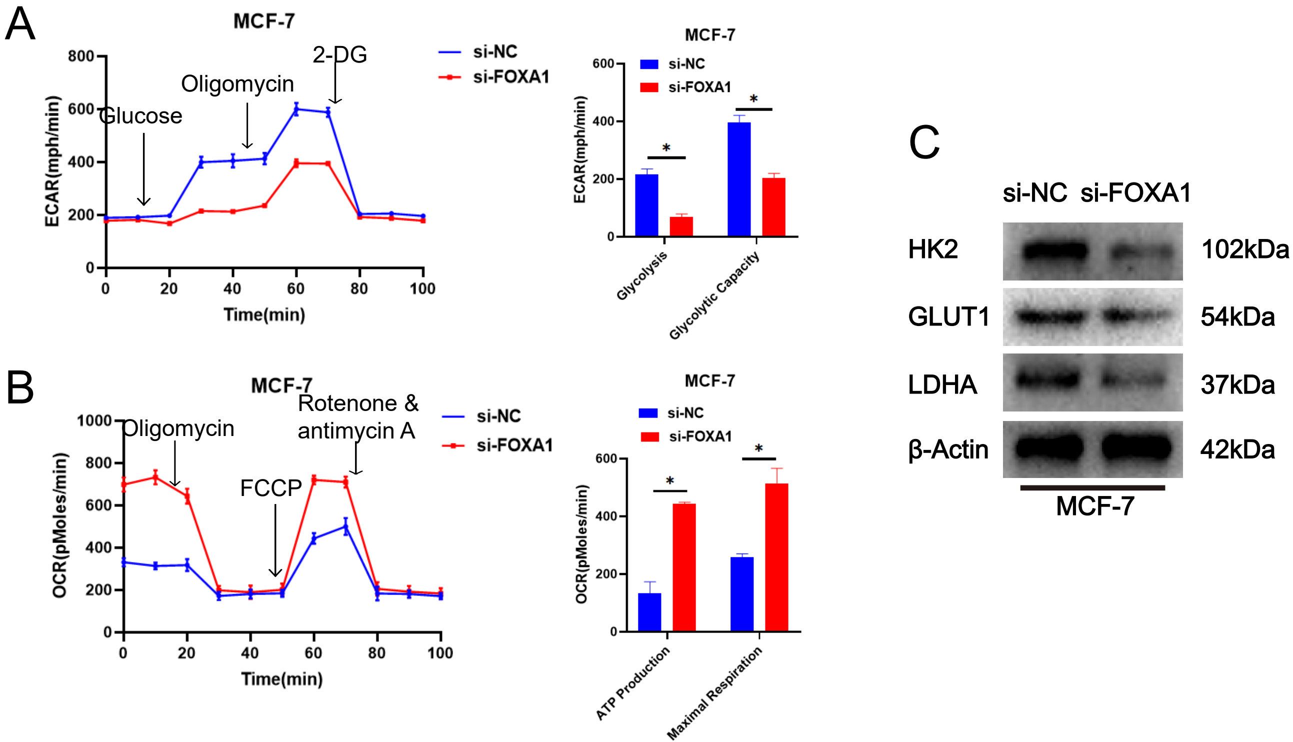
A
MCF-7
si-NC
si-FOXA1
Glucose
Oligomycin
2-DG
ECAR(mph/min)
Time(min)
MCF-7
si-NC
si-FOXA1
ECAR(mph/min)
Glycolysis
Glycolytic Capacity
B
MCF-7
Oligomycin
FCCP
Rotenone &
antimycin A
si-NC
si-FOXA1
OCR(pMoles/min)
Time(min)
MCF-7
si-NC
si-FOXA1
OCR(pMoles/min)
ATP Production
Maximal Respiration
C
si-NC
si-FOXA1
HK2
102kDa
GLUT1
54kDa
LDHA
37kDa
β-Actin
42kDa
MCF-7
